# *Saccharomyces cerevisiae* fermentation products (SCFP) stabilize the ruminal microbiota of lactating dairy cows during periods of a depressed rumen pH

**DOI:** 10.1186/s12917-020-02437-w

**Published:** 2020-07-11

**Authors:** Hein M. Tun, Shucong Li, Ilkyu Yoon, Sarah J. Meale, Paula A. Azevedo, Ehsan Khafipour, Jan C. Plaizier

**Affiliations:** 1 Department of Animal Science, University of Manitoba, Winnipeg, MB Canada; 2 Present address: HKU-Pasteur Research Pole, School of Public Health, Li Ka Shing Faculty of Medicine, The University of Hong Kong, Hong Kong, Hong Kong SAR; 3 Diamond V, Cedar Rapids, IA USA; 4 School of Agriculture and Food Science, University of Queensland Gatton campus, Gatton, Australia; 5 Present Address: Diamond V, Cedar Rapids, IA USA

## Abstract

**Background:**

Effects of *Saccharomyces cerevisiae* fermentation products (SCFP) on rumen microbiota were determined in vitro and in vivo under a high and a depressed pH. The in vitro trial determined the effects of Original XPC and NutriTek (Diamond V, Cedar Rapids, IA) at doses of 1.67 and 2.33 g/L, respectively, on the abundances of rumen bacteria under a high pH (> 6.3) and a depressed pH (5.8–6.0) using quantitative PCR (qPCR). In the in vivo trial eight rumen-cannulated lactating dairy cows were used in a cross-over design. Cows were randomly assigned to SCFP treatments (Original XPC, Diamond V, Cedar Rapids, IA) or control (No SCFP) before two 5-week experimental periods. During the second period, SCFP treatments were reversed. Cows on the SCFP treatment were supplemented with 14 g/d of SCFP and 126 g/d of ground corn. Other cows received 140 g/d ground corn. During the first 4 wk. of each period, cows received a basal diet containing 153 g/kg of starch. During week 5 of both periods, the rumen pH was depressed by a SARA challenge. This included replacing 208 g/kg of the basal diet with pellets of ground wheat and barley, resulting in a diet that contained 222 g/kg DM of starch. Microbial communities in rumen liquid digesta were examined by pyrosequencing, qPCR, and shotgun metagenomics.

**Results:**

During the in vitro experiment, XPC and NutriTek increased the relative abundances of *Ruminococcus flavefaciens*, and *Fibrobacter succinogenes* determined at both the high and the depressed pH, with NutriTek having the largest effect. The relative abundances of *Prevotella brevis*, *R. flavefaciens*, ciliate protozoa, and *Bifidobacterium* spp. were increased by XPC in vivo. Adverse impacts of the in vivo SARA challenge included reductions of the richness and diversity of the rumen microbial community, the abundances of Bacteroidetes and ciliate protozoa in the rumen as determined by pyrosequencing, and the predicted functionality of rumen microbiota as determined by shotgun metagenomics. These reductions were attenuated by XPC supplementation.

**Conclusions:**

The negative effects of grain-based SARA challenges on the composition and predicted functionality of rumen microbiota are attenuated by supplementation with SCFP.

## Background

Healthy and stable rumen microbiota are critical for maintaining efficient nutrient utilization, production and gut health of ruminants [1, 2]. However, the common practice of feeding diets with high grain and low coarse fiber contents to high-yielding dairy cows in order to meet their energy requirements can reduce gut health by inducing subacute ruminal acidosis (SARA) [2, 3]. This gut health disorder is defined as a reversible reduction of the rumen pH below 5.6 and 5.8 for a prolonged period [1, 4, 5]. This disorder can affect the composition and functionality of rumen microbiota by reducing the richness and diversity, reducing the abundances of protozoa and beneficial fibrolytic bacteria and by increasing the abundances of opportunistic pathogenic bacteria [3–5].

A variety of feed supplements are routinely used to enhance production efficiency and rumen function in cattle, including *Saccharomyces cerevisiae* fermentation products (SCFP). These products are rich in vitamins, minerals, oligosaccharides, organics acids, amino acids, peptides, antioxidants, and β-glucans that enhance the growth of bacteria [6], protozoa [7], and fungi [3] in the rumen. These bacteria include *Fibrobacter succinogenes* and *Ruminococcus albus* that are key digesters of structural carbohydrates in the rumen [2, 6].

Recent studies have demonstrated that supplementation with SCFP increases the efficiency of milk production and reduces adverse effects of SARA, such as the depression in rumen pH and milk fat [8, 9]. These effects of SCFP may be mediated through their effects on the composition and functionality of rumen microbiota. However, the underlying mechanisms have not yet been sufficiently explored. We, therefore, hypothesized that supplementing with SCFP reduces the adverse impacts of SARA on the composition and functionality of rumen microbiota. This hypothesis was tested in an in vitro and an in vivo trial by determining the effects of SCFP supplementation on microbiome composition and functionality during periods of high and depressed rumen pH.

## Results

### In vitro trial

**Fig. 1:**
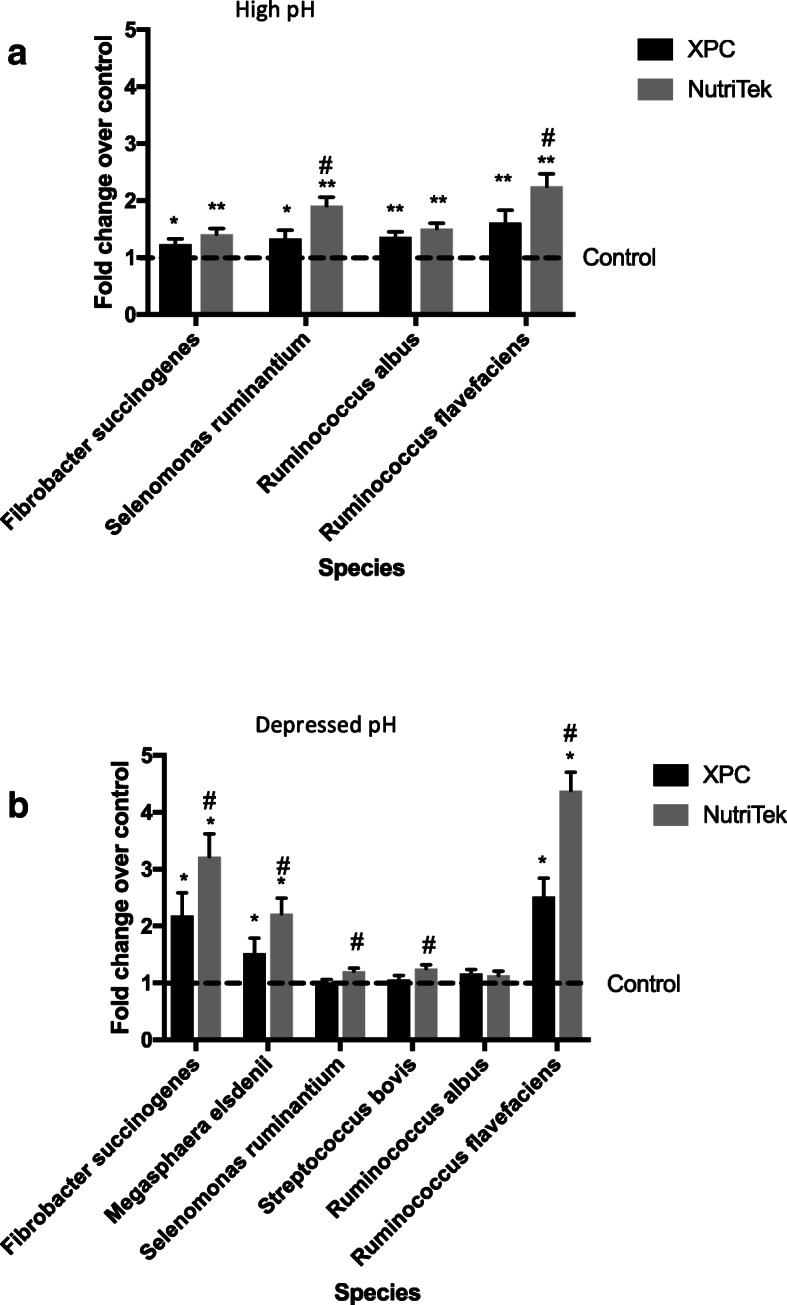
Fold change compared to the control treatment in populations of selected bacteria due to XPC or NutriTek supplementation determined by qPCR in vitro under a (**a**) high pH (> 6.3) or (**b**) depressed pH (5.8–6.0) by qPCR. * = XPC or NutriTek differ from control (*P* < 0.05). # = XPC and NutriTek differ (*P* < 0.05)

At the high pH, both XPC and NutriTek increased (*P* < 0.05) the relative abundances of *R. albus*, *R. flavefaciens*, *F. succinogenes*, and *S. ruminantium* compared to the control treatment as determined by qPCR*,* with NutriTek having the larger increase on *R. flavefaciens* (2.23 vs. 1.71 times, *P* < 0.05) and *S. ruminantium* than XPC (1.84 vs. 1.44 times, *P* < 0.05) (Fig. 1a). At the high pH, *M. elsdenii* was not detected. At the depressed pH (5.8–6.0), both XPC and NutriTek increased (*P* < 0.05) the relative abundances of *R. flavefaciens*, *F. succinogenes*, and *M. elsdenii* as determined by qPCR compared to the control treatment *(*Fig. 1b). These increases were larger for NutriTek than for XPC for *F. succinogenes* (3.15 vs. 2.23 times, *P* < 0.05), *M. elsdenii* (2.11 vs. 1.57 times, *P* < 0.05), *S. bovis* (1.38 vs. 1.25, *P* < 0.05), and *R. flavefaciens* (4.21 vs. 2.75 times, *P* < 0.01). However, at the depressed pH, the relative abundances of *R. albus*, *S. ruminantium* and *S. bovis* were not affected by supplementation with SCFP.

### In vivo trial

#### Richness, diversity and composition of rumen microbiota based on 16S rRNA gene sequencing

**Fig. 2:**
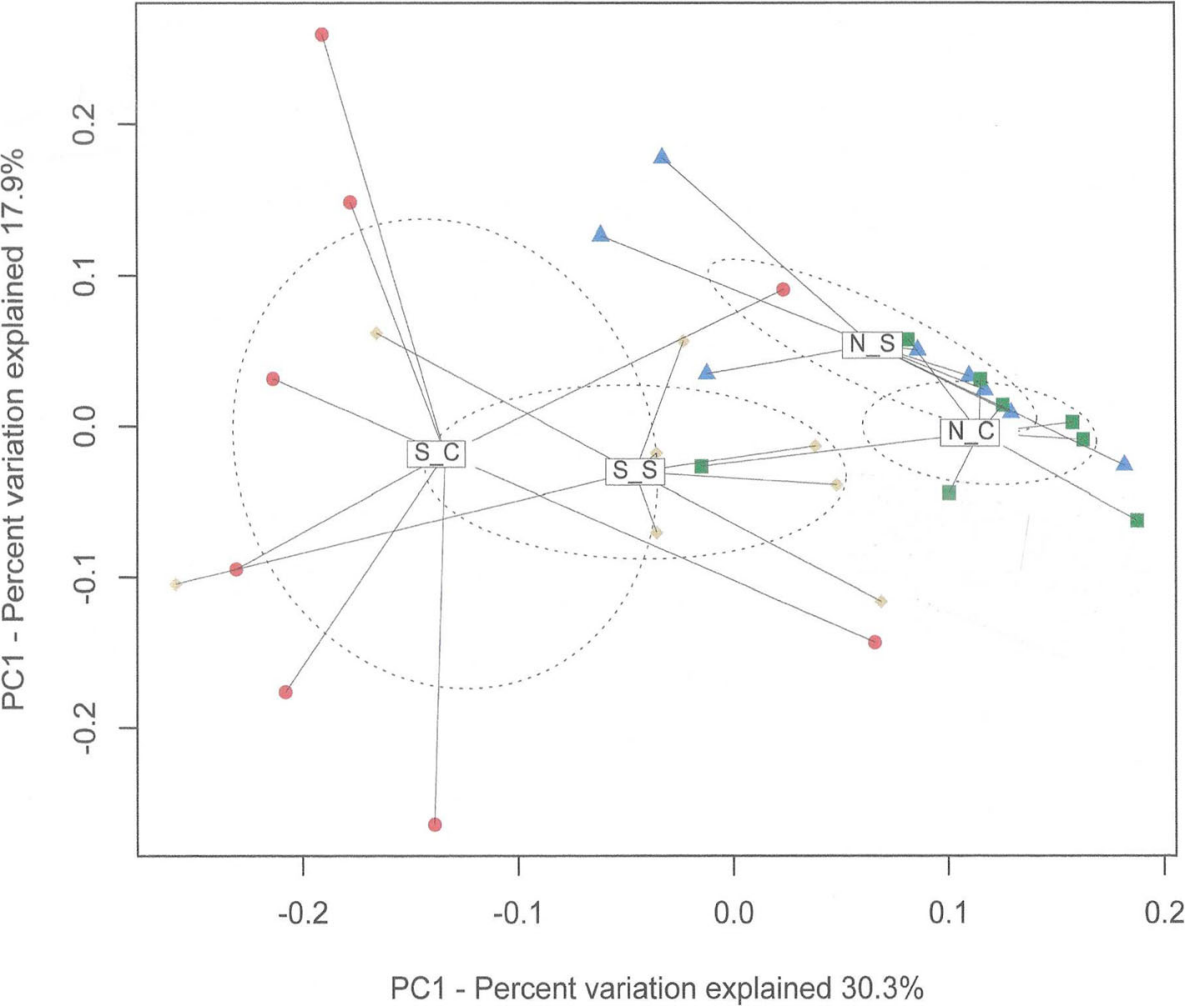
Two-dimensional PCoA plot based on weighted UniFrac distance matrix illustrates variation in rumen bacterial communities affected by subacute ruminal acidosis (SARA) challenge and supplementation of *Saccharomyces cerevisiae* fermentation products (SCFP). The significance of the effects of SARA challenge, SCFP supplementation and their interaction were *P* = 0.001, *P* = 0.64, and *P* = 0.05, respectively. Abbreviations in figure: N_C, normal diet without SCFP supplementation; N_S, normal diet with SCFP supplementation; S_C, SARA challenge without SCFP supplementation; S_S, SARA challenge with SCFP supplementation

The SARA challenge reduced all of the richness and diversity measures that were determined, with the exception of the number of reads (Table 1). The SCFP supplementation attenuated (*P* < 0.05) this reduction for the Chao1 (22 vs. 69, *P* < 0.001), ACE (167 vs. 480, *P* < 0.001), and Shannon indices (0.35 vs. 0.97, *P* < 0.001) and tended to limit (0.01 vs. 0.04, *P* < 0.10) this reduction for the Simpson index. The principal coordinate analysis plot based on weighted UniFrac distances showed that the SARA challenge affected the beta-diversity of the rumen microbial community, but that this effect was reduced by the supplementation with SCFP (Fig. 2). The effects of the SARA challenge on the relative abundances of phyla determined by 16S rRNA gene sequencing also depended on the SCFP treatment (Table 2). The SARA challenge only reduced the relative abundance of Bacteroidetes in the absence of SCFP (20.78 vs. 46.84%, *P* < 0.05), and only reduced the abundance of Chloroflexi (0.009 vs. 0.049%, *P* < 0.05) when SCFP was supplemented. Across SCFP treatments, the SARA challenge increased (*P* < 0.05) the relative abundances of Firmicutes (52.1 vs. 39.3%, *P* < 0.05) and Proteobacteria (9.7 vs. 1.0%, *P* < 0.05), reduced those of Spirochaetes (0.4 vs.1.6%, *P* < 0.01), Tenericutes (0.8 vs.1.8%, *P* < 0.01), and Cyanobacteria (0.4 vs. 0.8%, *P* < 0.05). SCFP supplementation tended to reduce the relative abundance of Spirochaetes (0.8 vs. 1.2%, *P* = 0.06) across normal feeding and SARA challenge.

**Table 1:** Effects of a *Saccharomyces cerevisiae* fermentation product (SCFP) and a subacute ruminal acidosis (SARA) challenge on richness and diversity indices of rumen bacterial communities calculated from 16S rRNA gene sequencing

Item	No SCFP		SCFP		SEM^1^		***P***-value	
	Control	SARA	Control	SARA		SARA	SCFP	SARA *SCFP
Number of Reads	2557	3927	3126	4697	833	0.1	0.44	0.91
Observed Species	197a	128b	196a	174b	15	0.01	0.14	0.12
Chao1	741^a^	337^b^	643	548	59	< 0.001	0.45	0.01
ACE	839^a^	359^b^	779	612	75	< 0.001	0.3	0.02
Shannon	5.88^a^	4.91^b^	5.88	5.53	0.19	< 0.001	0.17	0.05
Simpson	1.97^x^	1.93^y^	1.98	1.97	0.02	0.01	0.2	0.08
InvSimpson	63^a^	22^b^	65^a^	50^b^	5	0.01	0.22	0.19

^1^*SEM* standard error of mean

^a, b^LSmeans with different letters within SCFP treatment (No SCFP or SCFP) differ (*P* < 0.05)

^x, y^LSmeans with different letters within SCFP treatment (No SCFP or SCFP) tend to differ (*P* < 0.10)

**Table 2:** Relative abundances of ruminal bacterial phyla affected by *Saccharomyces cerevisiae* fermentation products (SCFP) and a subacute ruminal acidosis (SARA) challenge as determined by 16S rRNA gene sequencing

Phylum	No SCFP		SCFP		SEM^**1**^	P***-value***		
	Control	SARA	Control	SARA		SARA	SCFP	SARA* SCFP
Above 0.1% community								
Bacteroidetes	46.84^a^	20.78^b^	40.57	32.49	4.72	< 0.01	0.44	0.02
Firmicutes	35.08	53.86	43.44	50.25	5.06	0.04	0.69	0.3
Proteobacteria	1.12	13.88	0.78	5.5	2.74	0.02	0.39	0.46
Spirochaetes	1.41^x^	0.11^y^	1.79^x^	0.69^y^	0.22	< 0.01	0.06	0.68
Tenericutes	1.85^x^	0.45^y^	1.84^x^	1.06^y^	0.24	< 0.01	0.24	0.22
Cyanobacteria	1.05^a^	0.41^b^	0.60^a^	0.44^b^	0.15	0.02	0.23	0.22
SR1	0.75	0.43	0.53	0.22	0.2	0.14	0.32	0.18
TM7	0.41	0.28	0.31	0.23	0.08	0.22	0.36	0.99
Between 0.01 and 0.1% of community								
Verrucomicrobia	0.23a	0.02b	0.36a	0.04b	0.05	< 0.01	0.17	0.74
Actinobacteria	0.052	0.072	0.047	0.032	0.029	0.94	0.51	0.6
Chloroflexi	0.005	0.016	0.049^a^	0.009^b^	0.01	0.25	0.13	0.05
Elusimicrobia	0.033^a^	0.002^b^	0.042^a^	0.005^b^	0.013	0.02	0.8	0.87
Fibrobacteres	0.115	0.047	0.088	0.082	0.028	0.16	0.89	0.24
Lentisphaerae	0.095	0	0.042	0.011	0.021	0.16	0.89	0.24
Planctomycetes	0.021	0.016	0.046	0.008	0.013	0.13	0.54	0.26
Synergistetes	0.016	0	0.025	0.009	0.009	0.16	0.39	0.93

^1^*SEM* standard error of mean

^a, b^LSmeans with different letters within SCFP treatment (No SCFP or SCFP) differ (*P* < 0.05)

^x, y^LSmeans with different letters within SCFP treatment (No SCFP or SCFP) tend to differ (*P* < 0.10)

The relative abundances of *Succinivibrio* and SHD-231 were reduced by the SARA challenge, but these reductions were attenuated by supplementation with SCFP (Table S3). This challenge also affected the abundances of 10 out of the 16 genera with abundances above 1% of the community, and 19 of the 33 genera with abundances between 0.1 and 1% of the community. In particular, this challenge decreased the relative abundances of the *Paludibacter* (1.39 vs. 6.48%, *P* < 0.01), *Prevotella* (10.78 vs. 14.89%, *P* < 0.05), BF311 (0.21 vs. 0.89%, *P* < 0.01), CF231 (0.15 vs. 0.62%, *P* < 0.01) and YRC22 (0.23 vs. 0.66%, *P* < 0.01) within Bacteroidetes. Within the phylum Firmicutes, the SARA challenge increased the relative abundances of Butyrivibrio (9.40 vs. 5.77%, *P* < 0.05), *Shuttleworthia* (0.71 vs. 0.05%, *P* < 0.01), *Staphylococcus* (0.14 vs. 0.03%, *P* < 0.01), *Lactobacillus* (0.04 vs. 0.01%, *P* < 0.05) and *Acidaminococcus* (0.11 vs. 0.00%, *P* < 0.01), whereas it decreased these abundances on of L7A_E1 (0.00 vs. 0.04%, *P* < 0.01) and *Succiniclasticum* (0.66 vs. 1.59%, *P* < 0.01). The SARA challenge also reduced (*P* ≤ 0.04) the relative abundances of *Treponema* (0.40 vs. 1.58%, *P* < 0.01), *Pyramidobacter* (0.02 vs. 0.04%, *P* < 0.05) and *Sutterella* (0.01 vs. 0.20%, *P* < 0.01). Of the genera with relative abundances above 0.1% of the community, supplementation with SCFP only tended (*P* ≤ 0.07) to increase the abundance of *Treponema* (0.75 vs. 1.22%, *P* = 0.06) and of an unidentified genus within Firmicutes (2.18 vs. 4.77%, *P* = 0.07).

### Rumen microbiota abundance based on qPCR analysis

**Fig. 3:**
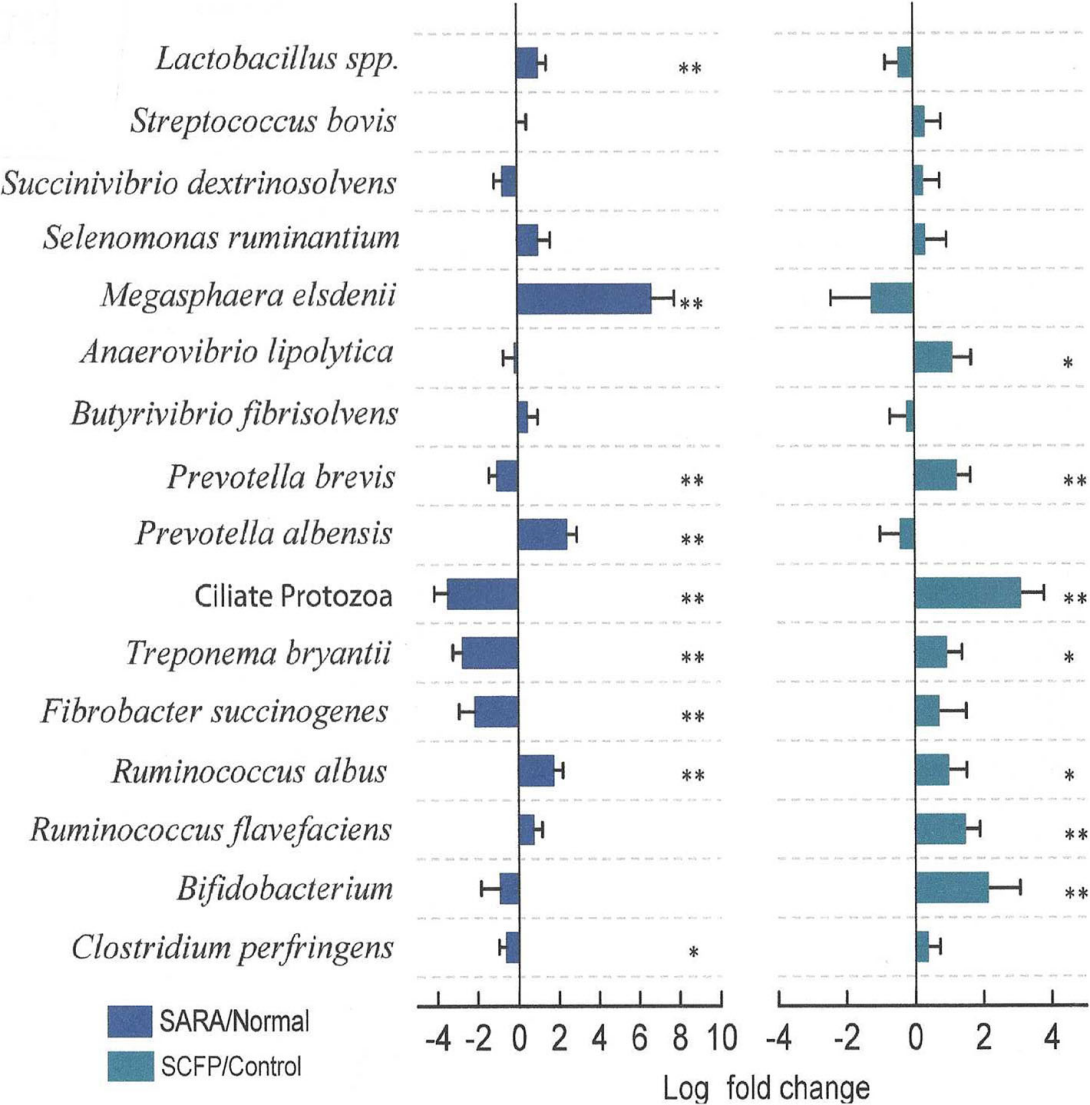
Effect of SCFP treatment and SARA challenge on the abundances of selected ruminal microorganisms, determined in vivo by qPCR. No interaction was detected; therefore, data was presented separately by SCFP treatment and SARA treatment. Comparisons are made SARA challenge vs. Normal Feeding and SCFP supplementation vs. no SCFP supplementation, * represents *P* < 0.10; ** represents *P* < 0.05

The effects of the SARA challenge and SCFP supplementation on the relative abundances of several ruminal bacterial species were determined using qPCR (Fig. 3). The challenge reduced the populations of *F. succinogenes* 2 log_2_ fold (*P* < 0.05), *Treponema bryantii *2.3 log_2_ fold (*P* < 0.05), *Prevotella brevis* (1 log_2_ fold, (*P* < 0.05), and ciliate protozoa 2 log_2_ fold (*P* < 0.05), and tended to reduce (*P* < 0.10) that of *Clostridium perfringens* 0.66 log_2_ fold (*P* < 0.01). In contrast, the SARA challenge increased (*P* < 0.05) the populations of *R. albus* 1.6 log_2_ fold (*P* < 0.05), *Prevotella albensis* 2.3 log_2_ fold (*P* < 0.05), *M. elsdenii* 6 log_2_ fold (*P* < 0.05), and *Lactobacillus* spp. 1 log_2_ fold (*P* < 0.05). Supplementation of SCFP increased (*P* < 0.05) the abundances of *Bifidobacterium* spp. 1.2 log_2_ fold (*P* < 0.5), *R. flavefaciens* 1.4 log_2_ fold (*P* < 0.05), *P. brevis* 1.2 log_2_ fold, and ciliate protozoa 3.2 log_2_ fold (*P* < 0.05), and tended to increase that of *R. albus* 1 log_2_ fold (*P* < 0.10), *T. bryantii* 0.7 log_2_ fold (*P* < 0.10) and *Anaerovibrio lipolytica* 1.1 log_2_ fold (*P* < 0.10)*.*

### Shotgun metagenomic analyses

Shotgun metagenomic sequencing showed that, across SCFP treatments, the SARA challenge tended to increase the relative abundance of Firmicutes (44.3 vs. 37.6%, *P* = 0.09), whereas it decreased those of Fibrobacteres (0.27 vs. 0.64%, *P* < 0.01), Spirochaetes (1.69 vs. 4.10%, *P* < 0.01), Tenericutes (2.95 vs. 4.73%, *P* < 0.01) and Verrucomicrobia (0.01 vs. 0.16%, *P* < 0.01) (Table 3). None of the abundances of the identified bacterial or eukaryote phyla were affected by SCFP, nor by the interaction between the SARA challenge and the SCFP treatments. In contrast, the relative abundance of archaea increased due to the SARA challenge in the absence of SCFP (1.15 vs. 0.71%, *P* < 0.05). However, the challenge reduced this abundance when SCFP was supplemented (0.70 vs. 1.80%, *P* < 0.05).

**Table 3:** Relative abundances of rumen microbial domains and phyla affected by *Saccharomyces cerevisiae* fermentation products (SCFP) supplementation and a subacute ruminal acidosis (SARA) challenge as determined by shotgun metagenomic-based analysis

Item	No SCFP		SCFP		SEM^**1**^		***P-value***	
	Control	SARA	Control	SARA		SARA	SCFP	SARA *SCFP
**Archaea**	**0.71**^**y**^	**1.15**^**x**^	**1.80**^**x**^	**0.70**^**y**^	**0.14**	**0.07**	**0.07**	**< 0.01**
Euryarchaeota	0.95	1.02	1.69a	0.92b	0.19	0.02	0.02	< 0.01
**Bacteria**	**98.36**	**97.52**	**97.05**	**97.36**	**0.7**	**0.72**	**0.34**	**0.42**
Actinobacteria	3.89	6.76	11.13	6.82	2.15	0.74	0.10	0.11
Bacteroidetes	42.06	36.51	34.59	40.2	4.49	0.99	0.68	0.22
Chlorobi	0	0.03	0	0	0	0.14	0.14	0.14
Fibrobacteres	0.69^a^	0.19^b^	0.59^a^	0.35^b^	0.14	0.01	0.82	0.35
Firmicutes	36.9^y^	46.49^x^	38.25^y^	42.01^x^	3.78	0.09	0.68	0.45
Fusobacteria	0.02	0	0	0	0.01	0.33	0.33	0.33
Nitrospirae	0.05	0.01	0.02	0	0.02	0.12	0.35	0.75
Planctomycetes	0.14^x^	0.05^y^	0.2^x^	0.11^y^	0.04	0.06	0.18	1
Proteobacteria	1.89	1.45	2	1.29	0.35	0.11	0.94	0.7
Spirochaetes	4.67^a^	1.52^b^	3.52^a^	1.86^b^	0.64	< 0.01	0.53	0.25
Synergistetes	0.01	0	0	0.01	0.01	1	1	0.18
Tenericutes	5.06^a^	2.95^b^	4.4^a^	2.14^b^	0.51	< 0.01	0.16	0.88
Verrucomicrobia	0.16^a^	0^b^	0.15^b^	0.01^b^	0.05	< 0.01	1	0.8
Unclassified	1.42	1.31	1.27	2.36	0.37	0.2	0.24	0.12
**Eukaryota**	**0.41**	**0.4**	**0.26**	**1.15**	**0.46**	**0.18**	**0.93**	**0.16**
Chlorophyta	0.04	0.01	0.02	0	0.02	0.21	0.52	1
Streptophyta	0.1	0.14	0.05	0.29	0.1	0.18	0.62	0.33
Unclassified	0.11	0.02	0.41	0.15	0.19	0.37	0.28	0.65

^1^*SEM* standard error of mean

^a, b^LSmeans with different letters within SCFP treatment (No SCFP or SCFP) differ (*P* < 0.05)

^x, y^LSmeans with different letters within SCFP treatment (No SCFP or SCFP) tend to differ (*P* < 0.10)

### Microbiome functions annotated by shotgun metagenomic sequencing

**Fig. 4:**
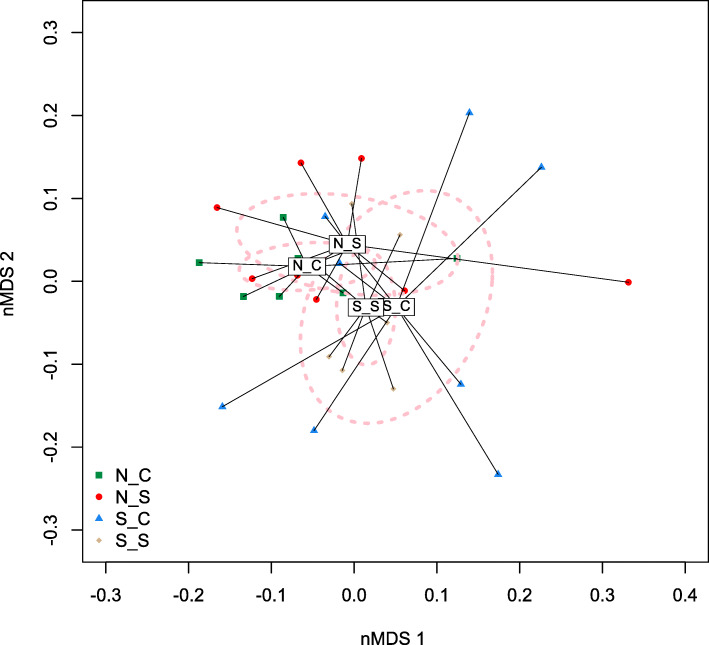
nMDS plot based on Bray-Curtis distance matrix illustrates variation in rumen bacterial communities affected by subacute ruminal acidosis (SARA) challenge and supplementation of *Saccharomyces cerevisiae* fermentation products (SCFP) from shotgun metagenomic sequencing. The significance of the effects of SARA challenge, SCFP supplementation and their interaction were *P* < 0.05, *P* < 0.05, and *P* < 0.42, respectively. Abbreviations in the figure: N_C, normal diet without SCFP supplementation; N_S, normal diet with SCFP supplementation; S_C, SARA challenge without SCFP supplementation; S_S, SARA challenge with SCFP supplementation

**Fig. 5:**
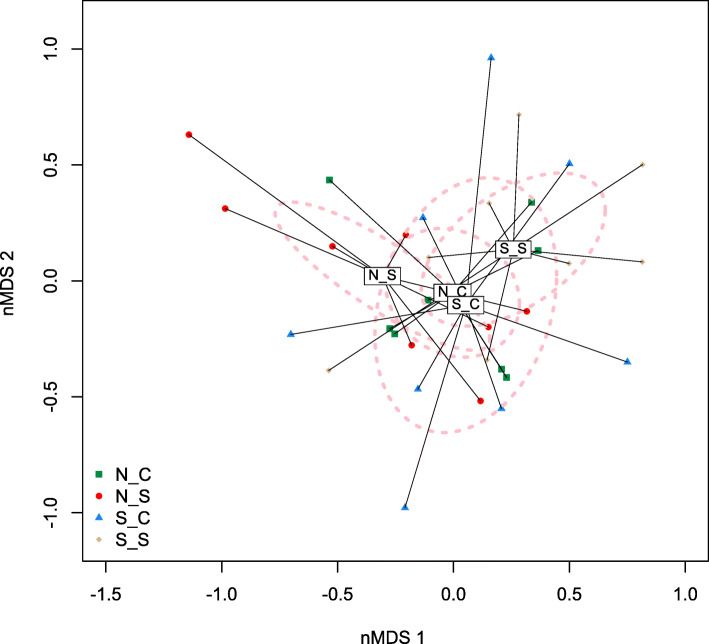
nMDS plot based on Bray-Curtis distance matrix of ruminal microbiome functions illustrates the effect of subacute ruminal acidosis (SARA) challenge and *Saccharomyces cerevisiae* fermentation products (SCFP) supplementation from shotgun metagenomic sequencing. The effects of SARA challenge, SCFP supplementation and their interaction were significant (*P* < 0.05) . Abbreviations in figure: N_C, normal diet without SCFP supplementation; N_S, normal diet with SCFP supplementation; S_C, SARA challenge without SCFP supplementation; S_S, SARA challenge with SCFP supplementation.

A total of 103.4 gigabytes (GB) of sequences were obtained from shotgun metagenomic sequencing, with an average of 10 M sequences per sample. The beta-diversity of the ruminal microbial community’s composition was affected by SCFP treatment and by the SARA challenge (Fig. 4). The ruminal microbial community’s functionality-based beta-diversity was also affected by SCFP (*P* < 0.05), but the impact of SCFP was greater during the SARA challenge than during normal feeding (Fig. 5). Functional annotations of the shotgun metagenomic sequences revealed that across SCFP treatments, the SARA challenge increased (*P* < 0.05) the “metabolism of aromatic compounds”, and tended (*P* < 0.10) to increase “protein metabolism” (Table 4). The SARA challenge only increased “sulfur metabolism” and increased “cell wall and capsule” in the absence of SCFP supplementation. The challenge only tended to reduce “nucleotides and nucleosides” when SCFP was supplemented. Supplementation with SCFP only increased “secondary metabolism” and “phosphorus metabolism” in the absence of SARA induction.

**Table 4:** Relative abundances of metabolic functions (based on subsystem annotation) as determined by shotgun metagenomic sequencing of the rumen microbiome as affected by *Saccharomyces cerevisiae* fermentation products (SCFP) supplementation and a subacute ruminal acidosis (SARA) challenge

Item	No SCFP		SCFP		SEM		***P-value***	
	Control	SARA	Control	SARA		SARA	SCFP	SARA *SCFP
Amino Acids and Derivatives	9.78	12.44	8.14	7.94	1.95	0.53	0.13	0.47
Carbohydrates	13.11	12.44	17.17	14.24	1.78	0.32	0.11	0.53
Cell Division and Cell Cycle	0.23	0.2	0.26	0.34	0.12	0.84	0.49	0.62
Cell Wall and Capsule	7.72	4.5	4.2	5.58	1.19	0.45	0.32	0.06
Clustering-based Subsystems	2.04	3.67	2.71	2.49	0.67	0.3	0.7	0.18
Cofactors, Vitamins, Prosthetic Groups, Pigments	2.52	3.5	2.98	2.8	0.5	0.43	0.81	0.25
DNA Metabolism	1.82	1.65	1.48	1.84	0.37	0.8	0.84	0.48
Dormancy and Sporulation	0.03	0.55	0.03	0.12	0.24	0.23	0.37	0.39
Fatty Acids, Lipids, and Isoprenoids	4.72	4.95	8.08	2.78	1.99	0.21	0.77	0.18
Iron Acquisition and Metabolism	0.34	0.57	0.79	0.34	0.22	0.62	0.64	0.13
Membrane Transport	0.39	0.72	0.79	0.62	0.17	0.63	0.39	0.16
Metabolism of Aromatic Compounds	0.7^b^	1.24^a^	0.58^b^	1.48^a^	0.03	0.04	0.86	0.59
Miscellaneous	2.39	3.26	3.03	2.56	0.54	0.72	0.96	0.23
Motility and Chemotaxis	0.08	0.25	0.13	0.51	0.18	0.13	0.39	0.57
Nitrogen Metabolism	1.7^x^	0.86^y^	1.18	1.39	0.26	0.24	0.99	0.06
Nucleosides and Nucleotides	0.55	0.63	1.05^x^	0.37^y^	0.19	0.13	0.53	0.06
Phages, Prophages, Transposable Elements, Plasmids	21.64	19.52	18.61	19.13	8.53	0.88	0.8	0.92
Phosphorus Metabolism	0.11	0.16	0.99^a^	0.14^b^	0.18	0.03	0.02	0.02
Photosynthesis	0.11^b^	0.18^a^	0.09^b^	0.19^a^	0.03	0.01	0.91	0.64
Potassium Metabolism	0.05	0.07	0.12	0.02	0.03	0.24	0.82	0.10
Protein Metabolism	16.18	16.69	11.71	19.18	2.19	0.08	0.66	0.12
Regulation and Cell Signaling	0.17	0.16	0.29	0.15	0.07	0.29	0.48	0.35
Respiration	6.08	4.67	5.72	7.38	0.91	0.89	0.21	0.10
RNA Metabolism	6.1	4.64	5.62	6.82	1.04	0.9	0.42	0.21
Secondary Metabolism	0.12	0.35	1.99	0.59	0.52	0.27	0.05	0.13
Stress Response	0.23	0.52	0.75	0.59	0.16	0.66	0.08	0.18
Sulfur Metabolism	0.31^b^	0.93^a^	0.54	0.44	0.17	0.15	0.47	0.05
Virulence, Disease and Defense	0.76	0.68	0.97	0.96	0.23	0.86	0.31	0.88

^1^*SEM* standard error of mean

^a, b^LSmeans with different letters within SCFP treatment (No SCFP or SCFP) differ (*P* < 0.05)

^x, y^LSmeans with different letters within SCFP treatment (No SCFP or SCFP) tend to differ (*P* < 0.10)

**Fig. 6:**
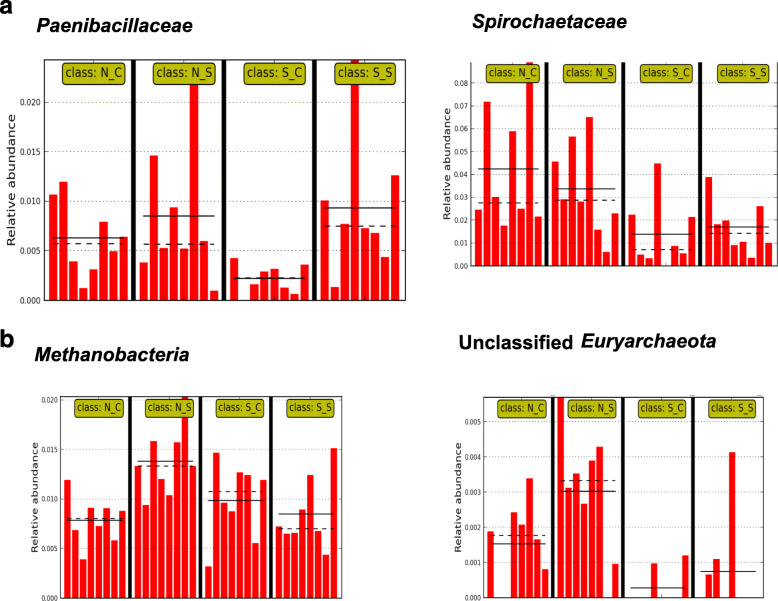
Microbial biomarkers identified from shotgun metagenomic data using linear discriminant analysis of effect size (LEfSe) using single class analysis (P < 0.05). **a** Paenibacillaceae and Spirochaetacaeae. **b***Methanobacteria* and Unclassified *Euryachaeota.* For all biomarkers, the effects of SARA challenge, SCFP supplementation and their interaction were significant (*P* < 0.05). Abbreviations in figure: N_C, normal diet without SCFP supplementation; N_S, normal diet with SCFP supplementation; S_C, SARA challenge without SCFP supplementation; S_S, SARA challenge with SCFP supplementation. Bars within treatment groups represent the relative abundances of the taxa in individual dairy cows (*n *= 8). The solid and dotted black lines represent the mean and median relative abundance values for each treatment group, respectively

**Fig. 7:**
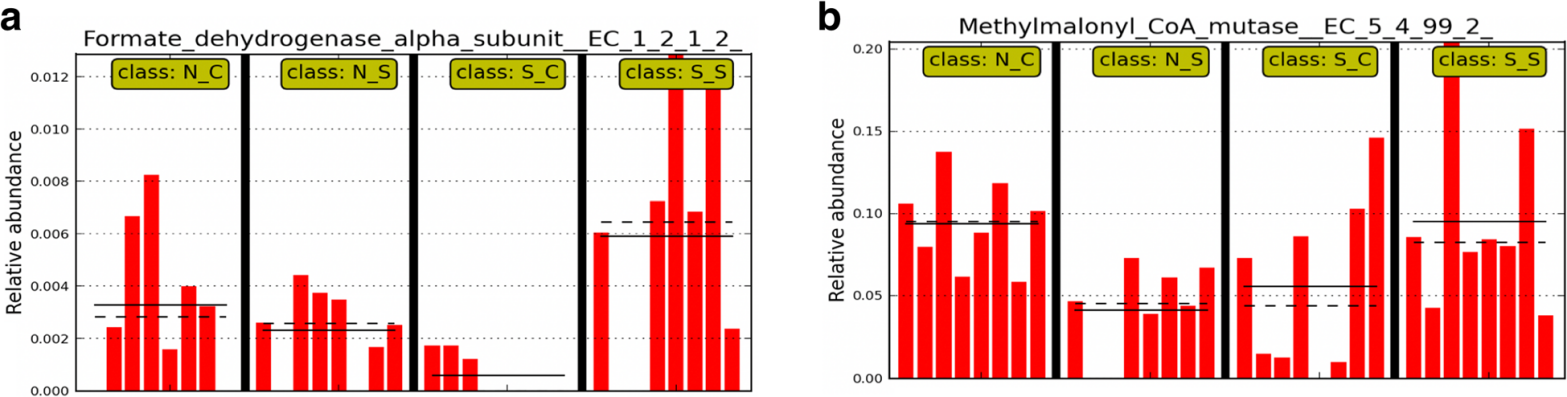
Microbial metabolic functions (**a**) formate dehydrogenase alpha (FDH) subunit EC.1.2.1.2 and (**b**) methylmalonyl CoA mutase EC.5.4.99.2, based on subsystem annotation as affected by SCFP supplementation during the SARA challenge (single class LEfSe, *P* < 0.05). For both enzymes, the effects of SARA challenge, SCFP supplementation and their interaction were significant (*P* < 0.05). Abbreviations in figure: N_C, normal diet without SCFP supplementation; N_S, normal diet with SCFP supplementation; S_C, SARA challenge without SCFP supplementation; S_S, SARA challenge with SCFP supplementation. Bars within treatment groups represent the relative abundances of the enzyme in individual dairy cows (*n *= 8). The solid and dotted black lines represent the mean and median relative abundance values for each treatment group, respectively

Using single class LEfSe analysis, two bacterial species belonging to unclassified Panibacillaceae (Fig. 6a) and Spriochaetaceae (Fig. 6a), and two archaeal taxa, *Methanobacteria* (Fig. 6b) and unclassified *Euryarchaeota* (Fig. 6b) were identified as microbial biomarkers that were affected by SCFP. The abundance of species belonging to Panibacillaceae and Spirochaetaceae were reduced by SARA challenge, but these reductions were alleviated by SCFP (*P* < 0.05). In contrast, the abundance of *Methanobacteria* and unclassified *Euryarchaeota* increased during the SARA challenge in cows that did not receive SCFP, whereas it decreased in cows that were supplemented with SCFP. Figure 7 shows that genes encoding for FDH and MCM increased due to SCFP supplementation during the SARA challenge, but not in the absence of SARA.

## Discussion

### SARA challenge

In order to conduct the SARA challenge, the dietary fibre content was reduced and that of starch increased. Hence, it was expected that this challenge would reduce abundances of fibrolytic and pH sensitive bacteria and increase those of amylolytic and pH tolerant bacteria, as well increase those of bacteria that utilize intermediates of starch fermentation [1, 2, 4]. In a parallel study [9], we showed that, across SCFP treatments, the SARA challenge increased the duration of the pH below 5.6 and reduced the acetate to propionate ratio in the rumen from 11.1 to 311.1 min/d, and from 3.07 to 1.74, respectively. The threshold used for SARA that was used in our study was a rumen pH depression below 5.6 of 180 min/d [1, 2]. Based on this threshold, SARA was induced successfully. This threshold was developed on the assumption that microbial enzymes and the growth of microorganisms in the rumen are sensitive to this pH depression, and that a more severe rumen pH depression increase the concentrations of serum amyloid A (SAA) in peripheral blood plasma and lipopolysaccharide endotoxin (LPS) in rumen fluid [1, 4]. In agreement, the parallel study also showed that, in the absence of SCFP supplementation, the SARA challenge increased the concentrations of SAA from 72.5 to 209.9 μg/mL and that of rumen LPS from 15,389 to 123,296 EU/mL. This endotoxin is shed by gram-negative bacteria, and can cause inflammation and disruption of the barrier function of the rumen epithelium [1, 4]. Hence, this increase in rumen LPS is additional evidence of the adverse effects of the SARA challenge on rumen bacteria.

The observed reductions in the microbial richness and diversity in the rumen during the SARA challenge agree with earlier research that shows that SARA compromises the functionality of the rumen microbiota [3, 10, 11]. Also, in agreement with previous studies [3, 10, 11], our gene sequencing data demonstrated that Bacteroidetes, Firmicutes and Proteobacteria were the dominant ruminal phyla. Members of the Bacteroidetes phyla are considered more efficient fermenters of complex polysaccharides, compared to Firmicutes, and thus, represent the primary degraders for these compounds in the rumen [12]. As such the reduction in the relative abundance of Bacteroidetes during the SARA challenge may be undesirable. Reductions in this abundance of Bacteroidetes and increases in the relative abundances of Firmicutes and Proteobacteria due to excessive grain feeding and grain-based SARA challenges have been reported previously [3, 13]. However, in contrast to earlier studies, our gene sequencing data did not show that the SARA challenge increased the relative abundances of Firmicutes [3, 10, 14] and Proteobacteria [15]. However, in contrast with our findings and using a similar sequencing technology, Fernando et al. [14] observed that switching cows from a low grain to a high grain diet increased the relative abundance of Bacteroidetes, but did not affect that of Proteobacteria. These discrepancies among studies may result from differences in the amount of dietary grain, and therefore starch, in the control and high grain treatments, as large differences between these treatments may result in larger responses. Responses to such increases also vary among cows. This was shown by Khafipour et al. [15], who demonstrated that a large increase in grain feeding increased the relative abundance of Firmicutes in the rumen in cows that responded severely to a grain-based SARA challenge, but not in cows that responded moderately to this challenge.

The ordination plots of the microbiome beta-diversity based on 16S rRNA gene sequencing and shotgun metagenomics also demonstrated that the SARA challenge affected the microbiological beta-diversity of the rumen.

The effects of the SARA challenge on the abundances of bacterial phyla determined with shotgun metagenomics differed from that determined by 16S rRNA gene sequencing. In contrast to the 16S rRNA gene sequencing data, the metagenomic data did not show effects of the SARA challenge, the SCFP treatment and their interaction on the abundances of Bacteroidetes or Proteobacteria. These discrepancies between methods may be due to differences in sequencing targets, biases and coverage, including unequal amplification of species’ 16S rRNA genes, insufficient depth of metagenomic sequencing for the identification of low abundance species, and insufficient taxa-specific microbial reference genome sequences between the techniques [16–18]. As a result, Shah et al. [16] recommended that, until sufficient reference sequences are available, metagenomic shotgun analysis needs to be combined with 16S rRNA gene sequencing to obtain sufficient accuracy on the effects of dietary treatments on the composition of gut microbiota.

In the rumen, Bacteroidales and *Prevotella* were the most abundant taxa within Bacteroidetes and several genera within Ruminococcaceae were the most abundant genera within Firmicutes, which agrees with previous studies [3, 10, 13]. The proportion of the rumen genera affected by the SARA challenge was higher in our study than that in earlier studies, and there is no agreement among studies on which genera are the most affected by a SARA challenge [10, 11]. Reasons for this discrepancy may be that the rumen pH depression in our study was greater than in previous studies, and that the response of rumen genera to grain challenge is highly variable. Nevertheless, our study and earlier studies agree that the number of genera affected by SARA challenges is limited.

Our study, as well as earlier studies using qPCR, agree that SARA challenges affect the populations of many species of rumen bacteria. Whereas in agreement with previous studies [3, 10, 13], the SARA challenge increased the populations of starch and sugar utilizing bacteria, this challenge only reduced the populations of fibrolytic bacteria in the absence of SCFP. The reduction in the populations of fibrolytic bacteria in the absence of SCFP agrees with earlier studies [3, 13, 15]. Hence, at the species level, the changes in the size of these populations appear to reflect changes in available substrates. However, as rumen bacteria compete for substrates and share functionality, assessing the populations of only a selected few species of rumen bacteria may not provide a comprehensive overview of changes in the species composition of rumen microbiota [10, 11, 19].

The analysis of the relative abundances of metabolic functions by shotgun metagenomic sequencing showed that the SARA challenge increased or tended to increase protein metabolism (*P* = 0.08), and metabolism of aromatic compounds (*P* = 0.04). The challenge tended to decrease nitrogen metabolism (*P* < 0.1) and increased sulphur metabolism (*P* < 0.05) in the absence of SCFP supplementation. In the rumen, nitrogen metabolism primarily involves microbial proteolytic activity [20, 21]. The challenge only decreased phosphorus metabolism (*P* < 0.05) when SCFP were supplemented. This activity of proteolytic microbes depends on the chemical structure of dietary proteins, the rumen pH, and the predominant proteolytic species of bacteria present in the rumen [21]. Thus, the influence of the SARA challenge on nitrogen metabolism in the rumen may be a consequence of changes in the abundance of proteolytic bacteria, which may be driven by a reduction of the rumen pH [2].

The impact of the reductions in the abundances of Paenibacillaceae and Spirochaetaceae by the SARA challenge is unclear. The family Spirochaetaceae is associated with diseases in ruminants with the rumen and feces being considered a reservoir for these microbes [22]. The family Paenibacillaceae are organoheterotrophic, and utilize carbohydrates as well as amino acids [23].

The reductions of the abundances of the genes coding for the enzymes MCM and FDH that resulted from the SARA challenge may impair the functionality of rumen microbiota. The enzyme FDH catalyzes the oxidation of formate to CO_2_, and has been found in bacteria [17] and hydrogenotrophic methanogenic archaea [24]. As a result, many species of these archaea utilize formate instead of H_2_. A reduced expression of FDH may, therefore, enhance H_2_ utilization, and, lessen the decline in pH during a SARA challenge. In contrast, MCM catalyzes the conversion of methylmalonyl-CoA to succinyl-CoA, and is therefore, part of key metabolic pathways [25]. In humans, it is the first vitamin B12-depedent enzyme, and a deprivation of its activity results in metabolic acidosis and methylmalonic acidemia [26]. Little is known about the role of this enzyme in ruminants, but due to its critical role in metabolism, a reduced expression of the MCM gene may be considered adverse.

The SARA challenges resulted in many changes in the composition and predicted functionality of the rumen microbiota. However, it is difficult to conclude if these changes are normal adaptations to a higher grain diet, or if these changes affect the health of dairy cows adversely. However, as SARA challenges reduce feed intake, fibre digestion, and milk fat production, and increase in bacterial endotoxins in rumen digesta and blood plasma, as well as markers of inflammation [1, 4, 10], it may be assumed that, overall, these changes of the rumen microbiota are adverse.

### SCFP treatment

In both the in vitro and the in vivo trial, the qPCR data demonstrated that SCFP increased the populations of major fibrolytic and amylolytic bacteria, suggesting that the supplementation of SCFP is beneficial to the utilization of carbohydrates in the rumen [2]. In the in vivo trial, the populations of *F. succinongenes* and *R. flavefaciens* increased more under the depressed pH than under the high pH, which is beneficial as the populations of these bacteria are reduced by high grain feeding [3, 10, 13]. However, the increases in *F. succinogenes*, and *M. elsdenii* observed in vitro, were not evident in vivo. These discrepancies may have been due to the higher concentrations of cellulolytic bacteria and lower protozoal concentrations in the in vitro study. Despite the similarity of the qPCR method that was used, our in vivo qPCR results differ from those of Mullins et al. [18], as these authors did not observe effects of SCFP supplementation on the populations of selected species and genera of rumen microbiota. These authors suggested that the lack of these effects may be due to the incomplete coverage of the rumen microbiota by their qPCR analysis, and that the functionality, rather than the taxonomic composition of rumen microbiota, was altered by the SCFP supplementation. Other reasons for this discrepancy may include differences in the main grain source between the studies. Whereas dry-rolled barley grain was used in our study, high moisture corn was the main grain source in the earlier study. The higher rumen degradability of high moisture corn compared to dry-rolled barley grain may have resulted in differences in the rumen environment between these studies. This discrepancy highlights that factors other than the dietary starch content affect the taxonomic composition of rumen microbiota as determined by qPCR.

Our in vivo qPCR data also showed that SCFP supplementation increased the populations of *Bifidobacterium* spp. and ciliate protozoa. *Bifidobacterium* possess unique pathways to ferment non-structural carbohydrates [27]. Hence, an increase in the population of these bacteria will enhance rumen fermentation, especially when high-grain diets are fed. Protozoa stabilize the rumen environment by modulating bacterial metabolism and reducing the rate of fermentation of starch [28]. Hence, the attenuation of the SARA-induced reduction of the population of ciliate protozoa in the rumen due to supplementation with SCFP benefits rumen function during high grain feeding.

Many results of our study showed that the impacts of the SARA challenge on the composition and functionality of rumen microbiota were attenuated by the supplementation with SCFP. These effects of SCFP include limiting the SARA-induced reductions in the richness and diversity and the changes in the beta-diversity of rumen microbiota. Members of the rumen microbiota vary in their preferred substrates and functionality [29]. Hence, a larger richness and diversity of rumen microbiota allows more efficient use of resources under different conditions, including during nutritional challenges [2, 3, 29]. Hence, the limitation of the SARA-related drop of the richness and diversity of the rumen microbiota due to SCFP supplementation is beneficial to the health and production of the host cows.

Despite of the SARA-mitigating effects of SCFP, including effects on the abundances of several species of rumen bacteria, this supplementation only reduced the impact of SARA on one bacterial phylum, i.e. Bacteroidites, and one bacterial genus, i.e. unclassified *Bacteriodales*. Also, the abundance of only one genus, i.e. other Firmicutes, tended to be increased by SCFP supplementation. This raises the question why SCFP supplementation has so many more effects on the species than on the phylum and genus levels. This may, as suggested by Mullins et al. [18] be due to an incomplete coverage of the rumen microbiota in the qPCR, analysis, but other differences between qPCR and 16S rRNA sequencing may also play a role. Hence, it would be beneficial to confirm the qPCR results with 16S rRNA sequencing, but unfortunately the latter technique is currently not sufficient accurate for taxa identification at the species level.

Our results at the genus level, also contrasts recent findings that reported a decline in *Prevotella* and an increase in *Butyrivibrio* with SCFP supplementation in calves [30]. Yet, despite the proteolytic activity of *Prevotella* and butyrate producing activity of *Butyrivibrio,* no differences in the rumen concentrations of VFA were observed due to SCFP supplementation in their study and in our parallel study [9]. The discrepancy could possibly be driven by differing primers between the two sequencing studies (V1-V3 compared to V3, respectively), animal age and basal diet, as the calves were still receiving a milk-based diet in the study of Xiao et al. [30].

At the microbial functionality level, our shotgun metagenomic analysis showed that, in the absence of SCFP, the potential metabolisms of phosphorus and sulphur increased and that of nitrogen tended to decrease during the SARA challenge. This decrease in nitrogen metabolism may be related to a reduced proteolytic activity from rumen microbiota [31, 32]. The effects of increases in phosphorus and sulphur metabolism in the rumen translated to changes in functionalities at the level of the host cow. However, as most changes in functionalities resulting from the SARA affect the production and health of cows negatively, it may be assumed that these changes in microbiome functionalities are adverse, and that the SCFP mitigating effects on these functionalities are, therefore, beneficial.

Supplementation with SCFP only increased the abundances of genes encoding for FDH and MCM during the SARA challenge. Increased expression of FDH may, therefore, enhance H_2_ utilization, and, potentially lessen the decline in pH during a SARA challenge [24]. Little is known about the role of MCM in ruminants, but due to its critical role in metabolism, an increased expression of the MCM gene may be considered beneficial to cows [25].

The reductions in the abundances of Paenibacillaceae and Spirochaetaceae caused by the SARA challenge were also attenuated by SCFP supplementation. The impact of the SARA-mitigating effects of SCFP on the abundances of these families is unclear. Supplementation with SCFP also reduced the abundances of *Methanobacteria* and unclassified *Euryarchaeota* during the SARA challenge, but increased these abundances in the absence of SARA. As these taxa include methanogens [24], their reduction has the potential to enhance the energetic efficiency of ruminal fermentation.

## Conclusions

Our study has shown that grain-based SARA challenges reduce the richness, diversity, relative abundance of Bacteroidetes, and that of populations of several fibrolytic bacteria and protozoa. The challenge also increased the relative abundance of Firmicutes, the Firmicutes to Bacteroidetes ratio, and populations of several amylolytic bacteria, and altered the functionality of the ruminal microbiome as determined by shotgun metagenomics. These changes reduce the health and production of dairy cows. Supplementation with SCFP reduced several effects of the SARA challenges by increasing the populations of major fibrolytic and amylolytic bacteria, ciliate protozoa, and *Bifidobacterium* spp., by attenuating the SARA-related reductions of the richness and diversity of rumen microbiota, by increasing the abundances of genes encoding for important enzymes, and possibly by increasing the energetic efficiency of rumen fermentation. This supplementation also affected the functionality of the rumen microbiota, but the impact of these effects on the health and production of the host cows is not clear. Overall, our results show that supplementation with SCFP) stabilize the ruminal microbiota of lactating dairy cows during periods of a depressed rumen pH.

## Methods

### In vitro study

This study comprised of one run at a high pH (> 6.3) and two runs at a low pH (5.8–6.0). Rumen fluid from two non-lactating cows fed a 50:50 mixture of forage and concentrate diet was collected, pooled and immediately moved to an anaerobic chamber. The rumen fluid was filtered through four layers of cheesecloth and buffered to 5% with a buffer containing 8.2 mM NaCl, 3.63 mM (NH_4_)_2_SO_4_, 40 mM KH_2_PO_4_, 0.5 mM CaCl_2_-2H_2_O, 0.5 mM MgSO_4_-7H_2_O, 0.004 mM Resazurin, and 110 mM NaHCO_3_ [31]. For the neutral pH, 50 mL conical tubes containing commercial SCFP products (0.05 g XPC or 0.07 g NutriTek; Diamond V, Cedar Rapids, IA) and substrate (50:50 total mixed ration) were filled with 30 mL of buffered rumen fluid and incubated at 37 °C for 12 h during continuous mixing. The low pH experiment was performed with the buffer containing 50% of the NaHCO_3_ and substrate amount 2.5-fold higher than that of the neutral pH experiment. Within each run, treatments were run in quintuplet. After fermentation, tube contents were spun down at 15,000 × *g* for 10 min and the pellets were subjected to DNA extraction and qPCR.

### In vivo study

Cows were housed in individual stalls in the large animal metabolism facility of the Glenlea Research Station, University of Manitoba, MB, Canada. Cows were fed ad libitum at 9:00 and had unlimited access to fresh water. All procedures and handing were approved by the Fort Garry Campus Animal Care Committee in accordance with the Canadian Council for Animal Care guidelines [32]. At the end of the study, the cows returned to the dairy herd of the research station.

The details of the in vivo study were described previously Li et al. [9]. Briefly, eight lactating, rumen cannulated Holstein cows between 65 ± 16 days in milk (mean ± SD) and 605 ± 60 kg of body weight were obtained from the dairy herd of the University of Manitoba and returned to this herd after the completion of the study. Cows were blocked based on their parity (primiparous vs. multiparous) and within block randomly assigned to one of two dietary treatments: (1) a basal diet plus 14 g/d of *S. cerevisiae* fermentation products (SCFP, Original XPC, Diamond V, Cedar Rapids, IA) mixed with 126 g/d ground corn, or (2) a basal diet plus 140 g/d ground corn only (Control). The SCFP additive was supplemented as a top dress once daily immediately after feed delivery at 9:00. Experimental periods consisted of 4 weeks of feeding a basal diet followed by a one-week grain-based SARA challenge. The basal diet contained 160 g/kg crude protein, 372 g/kg neutral detergent fibre, and 153 g/kg starch on a dry matter basis. The SARA challenge was conducted by replacing 208 g/kg of the control diet with pellets of ground wheat and barley (50:50 w/w), and resulted in a diet that contained 183 g/kg crude protein, 259 g/kg neutral detergent fiber and 222 g/kg of starch on a DM basis. Experimental periods were separated by a two-week washout period, during which the control diet was fed and no SCFP was supplemented.

Rumen fluid was sampled at 6 h after feed delivery on the second and fourth day of week 4 and 5 of each experimental period by collecting approximately 500 mL of rumen fluid from the ventral sac through the cannula. Samples were mixed thoroughly, strained through four layers of cheesecloth, snap-frozen in liquid nitrogen and stored at − 80 °C until further analysis, as described by Li et al. [9].

### DNA extraction

Prior to DNA extraction, rumen fluid samples were thawed at room temperature and kept on ice. Subsequently, 1 mL of sample was centrifuged at 15000 × *g* for 20 min, and the supernatant was removed without disturbing the sediment. Genomic DNA (gDNA) was extracted from the sediment using a ZR fecal DNA kit (Zymo Research Corp., Orange, CA, USA) that included a bead-beating step for the mechanical lysis of the microbial cells. The concentration and purity of isolated gDNA were determined using a NanoDrop 2000 spectrophotometer (ThermoFisher Scientific, Wilmington, DE, USA). DNA samples were then normalized to a final concentration of 20 ng/μL for sequencing and 2 ng/μL for qPCR.

### Pyrosequencing

Rumen DNA was pyrosequenced at the Research and Testing Laboratory (Lubbock, TX; http://www.Researchtesting.com) using the bacterial tag-encoded GS FLX- Titanium amplicon sequencing as described by Pu et al. [33]. Briefly, a mixture of Hot Start, HotStar high fidelity Taq polymerases, and Titanium reagents were used to perform a one-step PCR (35 cycles) with primer 28f (5′-GAGTTTGATCNTGGCTCAG-3′) and 519r (5′- GTNTTACNGCGGCKGCTG-3′), which covered the variable regions V1-V3 of the bacterial 16S rRNA genes.

### Shotgun metagenomic library construction and sequencing

A total of 50 ng of extracted DNA from each sample was used to construct a shotgun metagenomic library using Nextera DNA Library Preparation kits according to the manufacturer’s protocol (Illumina, San Diego, CA, USA). Briefly, DNA was tagmented by the Nextera transposome, cleaned and the purified tagmented DNA was amplified in 5 cycle PCR program to add index 1 and 2, and common adapters P5 and P7 for multiplexing. Subsequently, the library was purified using AMPure XP beads (Beckman Coulter Genomics, Danvers, MA, USA) providing a size selection to remove short library fragments. The purified library was quantified using high sensitivity Qubit assays (Fisher Scientific, Ottawa, ON, Canada), and quality controlled to check the size distribution using high sensitivity Bioanalyses assays (Aglient Technologies, Santa Clara, CA, USA). Equal concentrations of four shotgun libraries were pooled for a sequencing run. Based on the size distribution, DNA concentrations for a pooled library was calculated for expected cluster generation as recommended by Illumina and subjected for 300 bp paired-end sequencing on a Miseq platform (Illumina, San Diego, CA, USA) at the Gut Microbiome Laboratory, University of Manitoba, Winnipeg, Canada. All sequencing data were deposited into the European Nucleotide Archive (ENA) (http://www.ebi.ac.uk/ena) of European Molecular Biology Laboratory-European Bioinformatic Institute (EMBL-EBI) and can be accessed via accession number ERP117106.

### Bioinformatics analyses

Sequencing data were assigned to their respective samples using associated bar code sequences, and filtered using the default parameters in QIIME version 1.9 [34]. All sequences shorter than 200 bp, those containing any ambiguous nucleotide bases and/or a homopolymer length greater than 7 bp were removed from the dataset. Chimeric sequences were detected and removed using the UCHIME algorithm (USEARCH 6.1) with the Greengenes gold database as a reference [35]. An open reference-based OTU picking approach was implemented using the QIIME algorithm and the Usearch61 method46, with default parameters to cluster the sequences at the 97% sequence similarity level using the Greengenes database (v13.5) [36]. Sequences that failed to cluster were subsampled for de novo OTU picking. All picked OTUs were subsequently aligned by PyNAST [37], and a phylogenetic tree was built using the FastTree method [38] to calculate UniFrac distances within QIIME [39]. Taxonomy was assigned to OTUs using RDP classifiers via QIIME with a confidence threshold of 0.8 [40].

The phylogenetic tree was built after it was rooted based on an artificially added archaea outgroup into the data set. Subsequently the OTU table was generated by QIIME, and the mapping file was assembled into a Phyloseq object [41]. The same sequencing depth for all samples was used for alpha-diversity analysis and beta-diversity analysis using Phyloseq [42]. Standard alpha-diversity metrics were evaluated, including the Observed Richness, Shannon index, and Simpson index. In terms of beta-diversity analysis, UniFrac-based principal coordinates analysis (PCoA) as well as nonmetric multidimensional scaling (nMDS) plots using Bray-Curtis distance were conducted with Phyloseq. Permutational multivariate analyses of variance (PERMANOVA) [41] based on the same similarity matrix were used to test the effect of the SARA challenges, SCFP supplementation and their interaction on the beta-diversity of rumen bacterial communities.

### Shotgun metagenomic analysis

In total, 103.4 GB sequences were obtained from 32 shotgun metagenomic samples with an average of 10 M sequences per sample. Data analysis was carried out as previously described [43] with some modifications. Briefly, host genomic sequences were removed from the generated shotgun metagenomic reads using a reference bovine genomic database [44] prior to submission to the MG-RAST pipeline [45] for both taxonomic and functional annotations. Taxonomies were annotated against the Greengenes database and functional genes were annotated against SEED subsystems in the MG-RAST pipeline. For parameter settings in this analysis, a maximum e-value of 0.01, minimum percent identity of 50, minimum alignment length of 50 and a raw score maximum of 0.3 were applied. The microbial biomarkers affected by SARA challenge and SCFP treatment were examined using Linear Discriminant Analysis with Effect Size (LEfSe) [46]. This included the non-parametric factorial Kruskal-Wallis sum rank test, followed by linear discriminant analysis (LDA) to estimate the effect size of each differentially abundant feature. Alpha value for the factorial Kruskal-Wallis test was 0.05 and the threshold on the logarithmic LDA score for discriminative features was set at 2.0, so that features with at least 100-fold shifts were considered significant. During LEfSe analysis, multiple hypothesis testing for the effects of SARA challenges, SCFP supplementation, and their interaction were conducted based on both single class and multiclass analysis approach.

### Quantitative PCR analysis

#### In vitro study

qPCR was carried out with a CFX96 Real-Time PCR System (BioRad Laboratories, Hercules, CA, USA) using SsoFast EvaGreen Supermix (BioRad Laboratories). Each reaction contained 250 nM primers (900 nM for *Megasphaera elsdenii*) (Integrated DNA Technologies, Coralville, IA, USA) 10 μL of supermix and 2 ng/mL of DNA. Primers used for universal bacteria, *F. succinogenes*, *M. elsdenii*, *Ruminococcus albus*, *R. flavefaciens*, *Selenomonas ruminantium*, and *Streptococcus bovis* are listed in Supplementary Table S1. The two-step amplification conditions were as follows: 98 °C for 2 min, then 40 cycles of 98 °C for 2 s and 55 °C for 5 s.

#### In vivo study

qPCR was conducted using an AB 7300 system (Applied Biosystems, Foster City, CA, USA) and the SDS Software (version 1.3; Applied Biosystems, Foster City, CA, USA) as described previously Khafipour et al. [15]. The oligonucleotides were synthesized by University Core DNA Services (University of Calgary, AB, Canada). Each reaction mixture was run in triplicate in a volume of 15 μL. Amplification reactions were carried out with 7.5 μL Power SYBR green PCR master mix (Applied Biosystems, Foster City, CA, USA) mixed with the selected primer set using a final concentration of 450 nM. The amplification consisted of one cycle of 95 °C (10 min), 40 cycles of denaturation at 95 °C (15 s), and annealing/extension at 60 °C (1 min). The only exceptions were for ciliate protozoa, where annealing/extension steps of 63 °C (30 s)/72 °C (30 s) and 54 °C (30 s)/72 °C (1 min) were applied.

The final melting analysis was achieved by slow heating from 65 °C to 95 °C in order to assess the specificity of the prime sets (Applied Biosystems, Foster City, CA, USA). The change in the quantity of target species in a tested sample (SARA or SCFP sample) relative to the same target species in a calibrator sample (Normal sample or No-SCFP sample) was calculated after all data were normalized for Eubacteria using the bacterial 16S RNA gene primer sets, which detect all bacterial strains. The efficiency of the amplification of each primer set was calculated from the slope of the standard curve generated with pool DNA samples and listed in Supplementary Table S2.

### Other statistical analyses

The effects of the SARA challenge and SCFP supplementation on all microbial variables, relative abundance of microbial populations, diversity indices, and metabolic functions of microbiome were analyzed in SAS (Ver. 9.3, SAS Inst., Inc., Cary, NC). The experimental unit was individual cow. Data were analyzed using a crossover and repeated measures design with the PROC MIXED procedure of SAS. The model included period, diet (Control or SARA), treatment (NoSCFP or SCFP) and all two and three-way (when appropriate) interactions as fixed factors. If the interaction term was not statistically significant (*P* > 0.05), it was excluded from the final model. The effects of cow and cow × diet × treatment were considered random. The PDIFF option was applied to evaluate pairwise comparisons between weeks, treatments, and diet × treatment interaction. Measurements collected at different sampling times on the same cow were considered repeated measures and the AR1 covariance structure was followed as this resulted in the smallest setting Bayesian information criterion. The normality of distributions of error terms were tested using the Shapiro-Wilks statistics using Proc UNIVARIATE. If the error terms were not normally distributed, then the data were transformed by raising it to the power of lambda. The required lambda value was the value calculated by Box-Cox transformation analysis using the TRANSREG procedure of SAS.

Data of the in vitro study were expressed as fold changes over the control treatment, and the effects of the SCFP treatment (XPC or NutriTek) on these changes were tested using the MIXED procedure of SAS. These analyses were conducted for each of the bacterial species and by incubation pH (High or Depressed pH). The effects of treatment were considered fixed, whereas the effects of duplicate run in the model of the Low pH were considered random. For all statistical analyses, effects were declared significant at *P* < 0.05, and tendencies at *P* < 0.10 were discussed.

## Supplementary information

**Additional file 1: Table S1.** The primer sets used for quantitative PCR (qPCR) in the in-vitro study. **Table S2.** The primer sets used for quantitative PCR (qPCR) in the in-vivo study. **TableS3.** Relative abundances of bacterial phyla of microbial communities in the rumen affected by *Saccharomyces cerevisiae* fermentation products (SCFP) and subacute ruminal acidosis (SARA) challenge.

## Data Availability

All sequencing data are deposited into the European Nucleotide Archive (ENA) (http://www.ebi.ac.uk/ena) of European Molecular Biology Laboratory-European Bioinformatic Institute (EMBL-EBI) and can be accessed via accession number ERP117106.

## Abbreviations

BW: Body weight
CCAC: Canadian Council on Animal Care
DNA: Deoxyribonucleic acid
FDH: Formate dehydrogenase
HSD: Honest significance test
LDA: Linear discriminant analysis
LEfSe: Linear Discriminant Analysis with Effect Size
LPS: Lipopolysaccharide
MCM: Methylmalonyl-CoA mutase
nMDS: Nonmetric multidimensional scaling plots
OTU: Operational taxonomic unit
PCoA: Principal coordinates analysis
PERMANOVA: Permutational multivariate analyses of variance
qPCR: quantitative polymerase chain reaction
SARA: Subacute ruminal acidosis
SCFP: *Saccharomyces cerevisiae* fermentation products
VFA: Volatile fatty acids
