# Supplementary material for: Saccharomyces cerevisiae fermentation products (SCFP) stabilize the ruminal microbiota of lactating dairy cows during periods of a depressed rumen pH
Source: BMC Vet Res. 2020 Jul 11;16:237. doi: 10.1186/s12917-020-02437-w (PMC7353776; doi:10.1186/s12917-020-02437-w)
Supplement: Supplementary file 1 — Additional file 1: Table S1. The primer sets used for quantitative PCR (qPCR) in the in-vitro study. Table S2. The primer sets used for quantitative PCR (qPCR) in the in-vivo study. TableS3. Relative abundances of bacterial phyla of microbial communities in the rumen affected by Saccharomyces cerevisiae fermentation products (SCFP) and subacute ruminal acidosis (SARA) challenge. [file 12917_2020_2437_MOESM1_ESM.docx]

**Supplementary Table S1.** The primer sets used for quantitative PCR (qPCR) in the *in-vitro* study.

| Target organism | Primer | Sequence (5’-3) | Efficiency | Reference |
| --- | --- | --- | --- | --- |
| Universal Bacteria | F | CCTACGGGAGGCAGCAG | 1.99 | ^1^ |
| Universal Bacteria | R | ATTACCGCGGCTGCTGG | 1.99 | ^1^ |
| *Ruminococcus albus* | F | TGTTAACAGAGGGAAGCAAAGCA | 1.97 | ^2^ |
| *Ruminococcus albus* | R | TGCAFCCTACAATCCGAACTAA | 1.97 | ^2^ |
| *Ruminococcus flavefaciens* | F | CGAACGGAGATAATTTGAGTTTACTTAGG | 1.99 | ^3^ |
| *Ruminococcus flavefaciens* | R | CGGTCTCTGTATGTTATGAGGTATTACC | 1.99 | ^3^ |
| *Fibrobacter succinogenes* | F | GGAGCGTAGGCGGAGATTCA | 1.99 | ^4^ |
| *Fibrobacter succinogenes* | R | GCCTGCCCCTGAACTATCCA | 1.99 | ^4^ |
| *Megasphaera elsdenii* | F | AGATGGGGACAACAGCTGGA | 1.94 | ^2^ |
| *Megasphaera elsdenii* | R | CGAAAGCTCCGAAGAGCCT | 1.94 | ^2^ |
| *Selenomonas ruminantium* | F | GGCGGGAAGGCAAGTCAGTC | 1.96 | ^4^ |
| *Selenomonas ruminantium* | R | CCTCTCCTGCACTCAAGAAAGACAG | 1,96 | ^4^ |
| *Streptococcus bovis* | F | TTCCTAGAGATAGGAAGTTTCTTCGG | 1.98 | ^2^ |
| *Streptococcus bovis* | R | ATGATGGCAACTAACAATAGGGGT | 1.98 | ^2^ |

**Supplementary Table S2.** The primer sets used for quantitative PCR (qPCR) in the *in-vivo* study.

| Target taxon/species tested | Primer set | Primer sequences | length | A Temp | GC% | Amplicon size | Efficiency | | Source of primer |
| --- | --- | --- | --- | --- | --- | --- | --- | --- | --- |
| Domain bacteria | 341-357F | CCTACGGGAGGCAGCAG | 17 | 55.2 | 70.6 | 189 | 2.03 | ^1^ | |
|  | 518-534R | ATTACCGCGGCTGCTGG | 17 | 56.2 | 64.7 |  |  |  | |
| Lactic Acid Bacteria | Ulac16S1F | AGCAGTAGGGAATCTTCCA | 19 | 51.5 | 47.4 | 345 | 1.9 | ^5,6^ | |
|  | Ulac16S1R | ATTCCACCGCTACACATG | 18 | 51.1 | 50.0 |  |  |  | |
| Streptococcus bovis | SBovis2F | ATTCTTAGAGATAGGGTTTCTCTT | 24 | 64.0 | 33.3 | 109 | 1.99 | ^7^ | |
|  | SBovis2R | ACCTTATGATGGCAACTAACAATA | 24 | 64.0 | 33.3 |  |  |  | |
| Succinivibrio dextrinosolvens | SucDex1F | TAGGAGCTTGTGCGATAGTATGG | 23 | 57.4 | 47.8 | 174 | 1.92 | ^4^ | |
|  | SucDex1R | CTCACTATGTCAAGGTCAGGTAAGG | 25 | 58.4 | 48.0 |  |  |  | |
| Selenomonas ruminantium | SelRum1F | GGCGGGAAGGCAAGTCAGTC | 20 | 60.4 | 65.0 | 83 | 1.96 | ^4^ | |
|  | SelRum1R | CCTCTCCTGCACTCAAGAAAGACAG | 25 | 61.1 | 52.0 |  |  |  | |
| Megasphaera elsdenii | MegEls1F | GACCGAAACTGCGATGCTAGA | 21 | 57.7 | 52.4 | 129 | 1.85 | ^8^ | |
|  | MegEls1R | CGCCTCAGCGTCAGTTGTC | 19 | 58.2 | 63.2 |  |  |  | |
| Anaerovibrio lipolytica | AnaLip2F | TGGGTGTTAGAAATGGATTCTAGTG | 25 | 56.6 | 40.0 | 109 | 1.98 | ^4^ | |
|  | AnaLip2R | GCACGTCATTCGGTATTAGCAT | 22 | 56.7 | 45.5 |  |  |  | |
| Butyrivibrio fibrisolvens | ButFib1F | CGCATGATGCAGTGTGAAAAGCTC | 24 | 72.0 | 50.0 |  | 1.88 | ^7^ | |
|  | ButFib1R | CCTCCCGACACCTATTATTCATCG | 24 | 72.0 | 50.0 |  |  |  | |
| Prevotella brevis | PreBre1F | GCTTGCTTTTGAAGATGGCGAC | 22 | 58.8 | 50.0 | 153 | 1.85 | ^4^ | |
|  | PreBre1R | CATCCCTTAGCGATAAATCTTTGCT | 25 | 57.6 | 40.0 |  |  |  | |
| Prevotella albensis | ProAlb4F | GCGCCACTGACGCTGAAG | 18 | 58.3 | 66.7 | 110 | 1.86 | ^4^ | |
|  | ProAlb4R | CCCCAAATCCAAAAGGACTCAG | 22 | 56.6 | 50.0 |  |  |  | |
| Ciliate Protozoal | UPorCil1F | GCTTTCGWTGGTAGTGTATT | 20 | 50.2 | 20.0 | 234 | 1.92 | ^9^ | |
|  | UPorCil1R | CTTGCCCTCYAATCGTWCT | 19 | 50.4 | 47.4 |  |  |  | |
| Treponema bryantii | TrpBry1F | GAGAAACGCTTTGTGGTGACTGT | 23 | 59.5 | 47.8 | 122 | 1.95 | ^4^ | |
|  | TrpBry1R | CCTACATGCCCTTTACGCTCAAT | 23 | 58.7 | 47.8 |  |  |  | |
| Fibrobacter succinogenes | FibSuc4F | GGAGCGTAGGCGGAGATTCA | 20 | 58.7 | 60.0 | 97 | 1.99 | ^4^ | |
|  | FibSuc4R | GCCTGCCCCTGAACTATCCA | 20 | 58.5 | 60.0 |  |  |  | |
| Ruminococcus albus | RumAlb1F | CCCTAAAAGCAGTCTTAGTTCG | 22 | 54.3 | 45.5 | 176 | 1.97 | ^10^ | |
|  | RumAlb1R | CCTCCTTGCGGTTAGAACA | 19 | 53.8 | 52.6 |  |  |  | |
| Ruminococcus flavefaciens | RumFla1F | CGAACGGAGATAATTTGAGTTTACTTAGG | 29 | 57.5 | 34.5 | 132 | 1.99 | ^3^ | |
|  | RumFla1R | CGGTCTCTGTATGTTATGAGGTATTACC | 28 | 59.3 | 42.9 |  |  |  | |
| Bifidobacterium | g-Bifid-F | CTCCTGGAAACGGGTGG | 17 | 56.0 | 64.7 | 577 | 2.02 | ^11^ | |
|  | g-Bifid-R | GGTGTTCTTCCCGATATCTACA | 22 | 64.0 | 45.5 |  |  |  | |
| Clostridium perfringens | CPerf165F | CGCATAACGTTGAAAGATGG | 20 | 58.0 | 45.0 | 105 | 1.98 | ^12^ | |
|  | CPerf269R | CCTTGGTAGGCCGTTACCC | 19 | 62.0 | 63.2 |  |  |  | |

**Reference**:

1 Muyzer, G., de Waal, E. C. & Uitterlinden, A. G. Profiling of complex microbial populations by denaturing gradient gel electrophoresis analysis of polymerase chain reaction-amplified genes coding for 16S rRNA. *Applied and environmental microbiology* **59**, 695-700 (1993).

2 Stevenson, D. M. & Weimer, P. J. Dominance of Prevotella and low abundance of classical ruminal bacterial species in the bovine rumen revealed by relative quantification real-time PCR. *Applied microbiology and biotechnology* **75**, 165-174, doi:10.1007/s00253-006-0802-y (2007).

3 Denman, S. E. & McSweeney, C. S. Development of a real-time PCR assay for monitoring anaerobic fungal and cellulolytic bacterial populations within the rumen. *FEMS microbiology ecology* **58**, 572-582, doi:10.1111/j.1574-6941.2006.00190.x (2006).

4 Khafipour, E., Li, S., Plaizier, J. C. & Krause, D. O. Rumen microbiome composition determined using two nutritional models of subacute ruminal acidosis. *Applied and environmental microbiology* **75**, 7115-7124, doi:10.1128/AEM.00739-09 (2009).

5 Lan, Y. *et al.* Real-time PCR detection of lactic acid bacteria in cecal contents of eimeria tenella-lnfected broilers fed soybean oligosaccharides and soluble soybean polysaccharides. *Poultry science* **83**, 1696-1702 (2004).

6 Walter, J. *et al.* Detection of Lactobacillus, Pediococcus, Leuconostoc, and Weissella species in human feces by using group-specific PCR primers and denaturing gradient gel electrophoresis. *Applied and environmental microbiology* **67**, 2578-2585, doi:10.1128/AEM.67.6.2578-2585.2001 (2001).

7 Fernando, S. C. *et al.* Rumen microbial population dynamics during adaptation to a high-grain diet. *Applied and environmental microbiology* **76**, 7482-7490, doi:10.1128/AEM.00388-10 (2010).

8 Ozutsumi, Y., Tajima, K., Takenaka, A. & Itabashi, H. Real-time PCR detection of the effects of protozoa on rumen bacteria in cattle. *Current microbiology* **52**, 158-162, doi:10.1007/s00284-005-0266-9 (2006).

9 Sylvester, J. T., Karnati, S. K., Yu, Z., Morrison, M. & Firkins, J. L. Development of an assay to quantify rumen ciliate protozoal biomass in cows using real-time PCR. *The Journal of nutrition* **134**, 3378-3384 (2004).

10 Wang, R. F., Cao, W. W. & Cerniglia, C. E. PCR detection of Ruminococcus spp. in human and animal faecal samples. *Molecular and cellular probes* **11**, 259-265, doi:10.1006/mcpr.1997.0111 (1997).

11 Matsuki, T., Watanabe, K., Fujimoto, J., Takada, T. & Tanaka, R. Use of 16S rRNA gene-targeted group-specific primers for real-time PCR analysis of predominant bacteria in human feces. *Applied and environmental microbiology* **70**, 7220-7228, doi:10.1128/AEM.70.12.7220-7228.2004 (2004).

12 Wise, M. G. & Siragusa, G. R. Quantitative detection of Clostridium perfringens in the broiler fowl gastrointestinal tract by real-time PCR. *Applied and environmental microbiology* **71**, 3911-3916, doi:10.1128/AEM.71.7.3911-3916.2005 (2005).

**Supplementary TableS3.** Relative abundances of bacterial phyla of microbial communities in the rumen affected by *Saccharomyces cerevisiae* fermentation product (SCFP) and subacute ruminal acidosis (SARA) challenge

| **Phylum** | **Family, Genus^1^** | **No SCFP** | |  | **SCFP** | | **SEM^2^** | **Significance (*P*-*value*)^3^** | | |
| --- | --- | --- | --- | --- | --- | --- | --- | --- | --- | --- |
|  |  | **Normal** | **SARA** |  | **Normal** | **SARA** |  | **SARA** | **SCFP** | **SARA *SCFP** |
|  |  | -------------------- above 1% population -------------------- | | | | | | | | |
| Bacteroidetes | Bacteroidales (o), Other | 1.63^a^ | 0.39^b^ |  | 1.69^a^ | 0.85^b^ | 0.19 | <0.01 | 0.21 | 0.32 |
| Bacteroidetes | Bacteroidales (o), Unclassified | 18.14^a^ | 7.80^b^ |  | 13.53^a^ | 11.65^b^ | 2.13 | 0.01 | 0.87 | 0.07 |
| Bacteroidetes | Porphyromonadaceae, *Paludibacter* | 6.43^a^ | 0.76^b^ |  | 6.53^a^ | 2.01^b^ | 1.07 | <0.01 | 0.61 | 0.67 |
| Bacteroidetes | Prevotellaceae, *Prevotella* | 15.51^a^ | 8.73^b^ |  | 14.27^a^ | 12.82^b^ | 2.31 | 0.04 | 0.44 | 0.16 |
| Bacteroidetes | S24-7, Unclassified | 1.96 | 2.24 |  | 1.68 | 3.65 | 0.68 | 0.17 | 0.50 | 0.30 |
| Firmicutes | Firmicutes (o), Other | 2.26 | 2.10 |  | 3.52 | 6.02 | 1.27 | 0.90 | 0.07 | 0.83 |
| Firmicutes | Clostridiaceae, Unclassified | 0.76^b^ | 1.07^a^ |  | 0.96^b^ | 1.79^a^ | 0.28 | 0.05 | 0.12 | 0.39 |
| Firmicutes | Lachnospiraceae, Other | 1.68^a^ | 3.49^b^ |  | 1.91^a^ | 3.51^b^ | 0.48 | <0.01 | 0.84 | 0.87 |
| Firmicutes | Lachnospiraceae, Unclassified | 1.07^b^ | 2.62^a^ |  | 1.08^b^ | 2.89^a^ | 0.61 | 0.04 | 0.87 | 0.87 |
| Firmicutes | Lachnospiraceae, *Butyrivibrio* | 4.53^b^ | 9.05^a^ |  | 7.01^b^ | 9.74^a^ | 1.17 | 0.02 | 0.32 | 0.57 |
| Firmicutes | Ruminococcaceae, Other | 4.32 | 7.65 |  | 5.77 | 3.60 | 1.61 | 0.95 | 0.77 | 0.17 |
| Firmicutes | Ruminococcaceae, Unclassified | 3.63^x^ | 4.97^y^ |  | 4.28^x^ | 6.41^y^ | 0.94 | 0.09 | 0.30 | 0.69 |
| Firmicutes | Ruminococcaceae, *Ruminococcus* | 7.21 | 7.86 |  | 10.60 | 4.07 | 2.37 | 0.27 | 0.94 | 0.16 |
| Firmicutes | Veillonellaceae, *Succiniclasticum* | 1.74^a^ | 0.42^b^ |  | 1.44^a^ | 0.89^b^ | 0.28 | <0.01 | 0.77 | 0.20 |
| Firmicutes | [Coprobacillaceae], *Sharpea* | 3.94 | 3.27 |  | 2.37 | 4.60 | 1.95 | 0.70 | 0.96 | 0.49 |
| Proteobacteria | Succinivibrionaceae, *Unclassified* | 0.37^b^ | 13.62^a^ |  | 0.15^b^ | 5.00^a^ | 2.69 | 0.01 | 0.38 | 0.42 |
|  |  | -------------------- between 0.1 and 1% population ------------------- | | | | | | | | |
| Bacteroidetes | BS11, Unclassified | 0.88^a^ | 0.20^b^ |  | 0.90^a^ | 0.22^b^ | 0.16 | <0.01 | 0.90 | 1.00 |
| Bacteroidetes | Bacteroidaceae, *BF311* | 0.27^a^ | 0.04^b^ |  | 0.21^a^ | 0.04^b^ | 0.05 | <0.01 | 0.63 | 0.58 |
| Bacteroidetes | Prevotellaceae, Other | 0.10^b^ | 0.22^a^ |  | 0.03^b^ | 0.19^a^ | 0.06 | 0.04 | 0.54 | 0.76 |
| Bacteroidetes | [Paraprevotellaceae], Unclassified | 0.26 | 0.10 |  | 0.33 | 0.33 | 0.07 | 0.35 | 0.10 | 0.38 |
| Bacteroidetes | [Paraprevotellaceae], *CF231* | 0.79^a^ | 0.10^b^ |  | 0.45^a^ | 0.19^b^ | 0.16 | 0.01 | 0.51 | 0.27 |
| Bacteroidetes | [Paraprevotellaceae], *YRC22* | 0.65^a^ | 0.12^b^ |  | 0.67^a^ | 0.34^b^ | 0.11 | <0.01 | 0.23 | 0.30 |
| Cyanobacteria | YS2 (o), Unclassified | 0.96^a^ | 0.16^b^ |  | 0.55^a^ | 0.24^b^ | 0.15 | <0.01 | 0.34 | 0.17 |
| Cyanobacteria | Streptophyta (o), Unclassified | 0.04^b^ | 0.26^a^ |  | 0.05^b^ | 0.20^a^ | 0.06 | <0.01 | 0.84 | 0.71 |
| Firmicutes | Clostridiales (o), Other | 0.56^y^ | 0.80^x^ |  | 0.62^y^ | 1.09^x^ | 0.19 | 0.07 | 0.36 | 0.54 |
| Firmicutes | Clostridiales (o), Unclassified | 0.30 | 0.24 |  | 0.28 | 0.45 | 0.08 | 0.53 | 0.28 | 0.21 |
| Firmicutes | Clostridiaceae, Other | 0.26 | 0.23 |  | 0.34 | 0.29 | 0.06 | 0.58 | 0.36 | 0.89 |
| Firmicutes | Lachnospiraceae, *Coprococcus* | 0.35 | 0.47 |  | 0.39 | 0.48 | 0.10 | 0.36 | 0.80 | 0.88 |
| Firmicutes | Lachnospiraceae, *Shuttleworthia* | 0.06^b^ | 0.59^a^ |  | 0.03^b^ | 0.82^a^ | 0.20 | <0.01 | 0.78 | 0.70 |
| Firmicutes | Lachnospiraceae, [*Ruminococcus*] | 0.09^b^ | 2.83^a^ |  | 0.07^b^ | 0.55^a^ | 0.72 | 0.02 | 0.63 | 0.66 |
| Firmicutes | Ruminococcaceae, *Oscillospira* | 0.09 | 0.10 |  | 0.15 | 0.13 | 0.04 | 0.86 | 0.28 | 0.74 |
| Firmicutes | Veillonellaceae, Other | 0.03^b^ | 0.23^a^ |  | 0.04^b^ | 0.11^a^ | 0.06 | 0.04 | 0.43 | 0.34 |
| Firmicutes | Veillonellaceae, Unclassified | 0.06^b^ | 0.44^a^ |  | 0.03^b^ | 0.27^a^ | 0.12 | 0.02 | 0.68 | 0.83 |
| Firmicutes | Veillonellaceae, *Megasphaera* | 0.00^y^ | 2.03^x^ |  | 0.00^y^ | 0.06^x^ | 0.52 | 0.06 | 0.63 | 0.63 |
| Firmicutes | Veillonellaceae, *Selenomonas* | 0.06 | 0.41 |  | 0.04 | 0.05 | 0.10 | 0.33 | 0.21 | 0.34 |
| Firmicutes | Coriobacteriales (o), Unclassified | 0.65 | 0.21 |  | 0.93 | 0.15 | 0.10 | <0.01 | 0.30 | 0.14 |
| Firmicutes | Coriobacteriaceae, Other | 0.18 | 0.46 |  | 0.10 | 0.23 | 0.09 | 0.01 | 0.15 | 0.74 |
| Firmicutes | Coriobacteriaceae, Unclassified | 0.09 | 0.31 |  | 0.16 | 0.09 | 0.07 | 0.48 | 0.54 | 0.06 |
| Firmicutes | Coriobacteriaceae, *Atopobium* | 0.39^y^ | 0.78^x^ |  | 0.51^y^ | 0.86^x^ | 0.17 | 0.06 | 0.60 | 0.91 |
| Firmicutes | Erysipelotrichaceae, *Bulleidia* | 0.14 | 0.15 |  | 0.13 | 0.22 | 0.04 | 0.29 | 0.53 | 0.42 |
| Proteobacteria | RF32 (o), Unclassified | 0.16 | 0.04 |  | 0.15 | 0.23 | 0.06 | 0.79 | 0.16 | 0.11 |
| Proteobacteria | Alcaligenaceae, *Sutterella* | 0.16^a^ | 0.00^b^ |  | 0.23^a^ | 0.02^b^ | 0.05 | <0.01 | 0.54 | 0.71 |
| SR1 | SR1 (p), Unclassified | 0.75 | 0.43 |  | 0.53 | 0.22 | 0.20 | 0.14 | 0.32 | 0.99 |
| Spirochaetes | Spirochaetaceae, *Treponema* | 1.39^a^ | 0.11^b^ |  | 1.77^a^ | 0.68^b^ | 0.22 | <0.01 | 0.06 | 0.70 |
| TM7 | F16, Unclassified | 0.41 | 0.28 |  | 0.31 | 0.23 | 0.08 | 0.22 | 0.36 | 0.74 |
| Tenericutes | Anaeroplasmataceae, *Anaeroplasma* | 0.65^a^ | 0.09^b^ |  | 0.47^a^ | 0.32^b^ | 0.12 | 0.01 | 0.52 | 0.18 |
| Tenericutes | Anaeroplasmataceae, *RFN20* | 0.77^a^ | 0.20^b^ |  | 0.68^a^ | 0.46^b^ | 0.13 | 0.01 | 0.52 | 0.18 |
| Tenericutes | RF39 (c), Unclassified | 0.32^a^ | 0.15^b^ |  | 0.57^a^ | 0.21^b^ | 0.09 | <0.01 | 0.14 | 0.39 |
| Verrucomicrobia | RFP12, Unclassified | 0.22^a^ | 0.02^b^ |  | 0.27^a^ | 0.02^b^ | 0.04 | <0.01 | 0.71 | 0.62 |
|  |  | -------------------- between 0.01% and 0.1% population -------------------- | | | | | | | | |
| Actinobacteria | Corynebacteriaceae, *Corynebacterium* | 0 | 0.072 |  | 0.014 | 0.004 | 0.015 | 0.27 | 0.33 | 0.12 |
| Actinobacteria | Micrococcaceae, *Kocuria* | 0.030^a^ | 0^b^ |  | 0.014^a^ | 0.002^b^ | 0.008 | 0.02 | 0.48 | 0.35 |
| Bacteroidetes | Bacteroidaceae, *Bacteroides* | 0.111 | 0.013 |  | 0.067 | 0.100 | 0.039 | 0.58 | 0.71 | 0.20 |
| Bacteroidetes | Rikenellaceae, Unclassified | 0.009 | 0.011 |  | 0.020 | 0.007 | 0.011 | 0.74 | 0.86 | 0.67 |
| Bacteroidetes | [Paraprevotellaceae], Other | 0.055 | 0.051 |  | 0.192 | 0.082 | 0.041 | 0.31 | 0.08 | 0.33 |
| Chloroflexi | Anaerolinaceae, *SHD-231* | 0.005 | 0.016 |  | 0.049 | 0.009 | 0.010 | 0.23 | 0.13 | 0.05 |
| Elusimicrobia | Elusimicrobiaceae, Unclassified | 0.033^a^ | 0^b^ |  | 0.042^a^ | 0.002^b^ | 0.011 | 0.01 | 0.81 | 0.88 |
| Fibrobacteres | Fibrobacteraceae, *Fibrobacter* | 0.115 | 0.046 |  | 0.088 | 0.082 | 0.028 | 0.17 | 0.90 | 0.24 |
| Firmicutes | Firmicutes (p), Other | 0.099 | 0.130 |  | 0.066 | 0.034 | 0.039 | 0.98 | 0.09 | 0.40 |
| Firmicutes | Staphylococcaceae, *Staphylococcus* | 0.033^b^ | 0.173^a^ |  | 0.026^b^ | 0.105^a^ | 0.033 | <0.01 | 0.34 | 0.46 |
| Firmicutes | Thermoactinomycetaceae, Unclassified | 0.012 | 0.050 |  | 0.007 | 0.022 | 0.020 | 0.37 | 0.71 | 0.79 |
| Firmicutes | Lactobacillaceae, *Lactobacillus* | 0.004^b^ | 0.046^a^ |  | 0.011^b^ | 0.031^a^ | 0.011 | 0.01 | 0.75 | 0.39 |
| Firmicutes | Streptococcaceae, *Streptococcus* | 0.056 | 0.027 |  | 0.078 | 0.028 | 0.024 | 0.13 | 0.66 | 0.69 |
| Firmicutes | Clostridia (c), Unclassified | 0.028 | 0.019 |  | 0.043 | 0.025 | 0.013 | 0.35 | 0.48 | 0.74 |
| Firmicutes | Catabacteriaceae, Unclassified | 0.094^a^ | 0.006^b^ |  | 0.125^a^ | 0.030^b^ | 0.027 | 0.01 | 0.38 | 0.92 |
| Firmicutes | Eubacteriaceae, *Anaerofustis* | 0 | 0.009 |  | 0.012 | 0.020 | 0.009 | 0.47 | 0.25 | 0.99 |
| Firmicutes | Eubacteriaceae, *Pseudoramibacter* | 0 | 0.039 |  | 0 | 0.008 | 0.009 | 0.22 | 0.48 | 0.48 |
| Firmicutes | Lachnospiraceae, *Anaerostipes* | 0.055 | 0.036 |  | 0.050 | 0.053 | 0.024 | 0.75 | 0.79 | 0.67 |
| Firmicutes | Lachnospiraceae, *Blautia* | 0.004 | 0 |  | 0.010 | 0.030 | 0.009 | 0.64 | 0.14 | 0.37 |
| Firmicutes | Lachnospiraceae, *Catonella* | 0^b^ | 0.039^a^ |  | 0^b^ | 0.005^a^ | 0.009 | 0.02 | 0.26 | 0.24 |
| Firmicutes | Lachnospiraceae, *Pseudobutyrivibrio* | 0.068 | 0.049 |  | 0.057 | 0.089 | 0.027 | 0.85 | 0.65 | 0.42 |
| Firmicutes | Ruminococcaceae, *Faecalibacterium* | 0.013 | 0.011 |  | 0 | 0.091 | 0.022 | 0.17 | 0.54 | 0.12 |
| Firmicutes | Veillonellaceae, *Acidaminococcus* | 0^b^ | 0.082^a^ |  | 0^b^ | 0.135^a^ | 0.036 | <0.01 | 0.94 | 0.93 |
| Firmicutes | Veillonellaceae, *Mitsuokella* | 0 | 0.047 |  | 0 | 0.023 | 0.014 | 0.11 | 0.55 | 0.55 |
| Firmicutes | Erysipelotrichaceae, *L7A_E11* | 0.023^a^ | 0b |  | 0.066^a^ | 0^b^ | 0.019 | 0.03 | 0.74 | 0.75 |
| Firmicutes | Erysipelotrichaceae, [*Eubacterium*] | 0.010 | 0.018 |  | 0 | 0.010 | 0.009 | 0.45 | 0.53 | 0.94 |
| Firmicutes | Erysipelotrichaceae, *p-75-a5* | 0.026 | 0.014 |  | 0.033 | 0.029 | 0.013 | 0.58 | 0.42 | 0.75 |
| Firmicutes | ML615J-28 (c), Unclassified | 0.031^a^ | 0^b^ |  | 0.039^a^ | 0.001^b^ | 0.012 | 0.03 | 0.89 | 0.92 |
| Lentisphaerae | Victivallaceae, Unclassified | 0.095^a^ | 0^b^ |  | 0.042^a^ | 0.011^b^ | 0.021 | 0.01 | 0.63 | 0.37 |
| Planctomycetes | Pirellulaceae, Unclassified | 0.021 | 0.016 |  | 0.046 | 0.008 | 0.013 | 0.13 | 0.54 | 0.26 |
| Proteobacteria | Other, Other | 0.005 | 0.007 |  | 0.020 | 0.017 | 0.010 | 0.98 | 0.31 | 0.84 |
| Proteobacteria | Rickettsiales (o), Unclassified | 0.178^a^ | 0^b^ |  | 0.083^a^ | 0.003^b^ | 0.039 | <0.01 | 0.65 | 0.60 |
| Proteobacteria | Desulfobulbaceae, *Desulfobulbus* | 0 | 0.045 |  | 0.037 | 0.041 | 0.020 | 0.29 | 0.49 | 0.37 |
| Proteobacteria | Gammaproteobacteria (c), Other | 0.010 | 0.051 |  | 0 | 0.034 | 0.016 | 0.11 | 0.65 | 0.86 |
| Proteobacteria | Succinivibrionaceae, Other | 0.025 | 0.041 |  | 0.037 | 0.066 | 0.034 | 0.61 | 0.67 | 0.87 |
| Proteobacteria | Succinivibrionaceae, *Succinivibrio* | 0.113 | 0.033 |  | 0.013 | 0.058 | 0.024 | 0.53 | 0.18 | 0.02 |
| Synergistetes | Dethiosulfovibrionaceae, *Pyramidobacter* | 0.016^a^ | 0^b^ |  | 0.025^a^ | 0.001^b^ | 0.008 | 0.04 | 0.54 | 0.67 |
| Tenericutes | Anaeroplasmataceae, Unclassified | 0.052 | 0.016 |  | 0.032 | 0.032 | 0.018 | 0.36 | 0.92 | 0.34 |
| Tenericutes | Mycoplasmataceae, Unclassified | 0.037 | 0 |  | 0.075 | 0.042 | 0.018 | 0.17 | 0.12 | 0.94 |
| Verrucomicrobia | WCHB1-25, Unclassified | 0 | 0 |  | 0.076 | 0.016 | 0.019 | 0.38 | 0.02 | 0.38 |
| WPS-2 | WPS-2 (p), Unclassified | 0 | 0 |  | 0.100^x^ | 0^y^ | 0.022 | 0.09 | 0.09 | 0.09 |

1. For the taxons unclassified on the family level, a higher level of classification are used. (p) signifies Phylum, (c) signifies Class, and (o) signifies Order.

2. SEM, the average of the standard errors across the treatments.

3. the statistical significance of SARA challenge across the treatments (SARA), SCFP supplementation across the treatments (SCFP), the interaction between SARA and SCFP (SARA*SCFP).
